# Multivariate Geostatistical Modeling of *Phytophthora rubi* and *Pratylenchus penetrans* in Red Raspberry Fields

**DOI:** 10.2478/jofnem-2025-0038

**Published:** 2025-09-24

**Authors:** J. B. Contina, D. R. Kroese, T. W. Walters, J. E. Weiland, I. A. Zasada

**Affiliations:** Driscoll’s Inc., Red Bluff, CA 96080; USDA Forest Service, Pacific Northwest Research Station, Corvallis, OR 97331; Walters Ag Research, Anacortes, WA 98221; USDA Agricultural Research Service, Horticultural Crops Research Unit, Corvallis, OR 97330

**Keywords:** ecology and epidemiology, *Phytophthora* root rot, quantitative epidemiology, root lesion nematode nematode, spatial autocorrelation, spatial autoregressive model

## Abstract

The soilborne pathogens *Phytophthora rubi* and *Pratylenchus penetrans* threaten commercial red raspberry production in the U.S. Pacific Northwest (Washington and Oregon). Due to these soilborne pathogens, the lifespan of raspberry fields has declined from over 10 years to 5–7 years. Management primarily revolves around pre-plant soil fumigation; however, regulations have made fumigation more difficult and expensive. Understanding the spatial distribution of soilborne pathogens may provide new insights into implementing targeted disease management. Therefore, this study assessed the distribution of disease severity, *P. rubi*, and *P. penetrans* in four raspberry fields in Oregon and Washington. Geostatistical analyses and spatial autoregressive modeling were performed to elucidate the interactions between soil physical characteristics and densities of *P. rubi* and *P. penetrans*, as well as disease severity, in infested fields. In general, disease severity and the pathogens were spatially clumped in the fields. Soil texture and field elevation did not consistently influence *P. rubi* and *P. penetrans* distributions in the field. Disease severity was mostly driven by *P. rubi* root infestation, and no significant interactions were found between *P. rubi* and *P. penetrans* in roots. This study aimed to provide a greater understanding of the ecology and epidemiology of soilborne pathogens in commercial red raspberry fields to enable the development of a targeted integrated pest management strategy.

The Pacific Northwest (PNW; Washington and Oregon) and California lead the commercial production of red raspberry (*Rubus idaeus*) in the United States. The combined producing area is estimated to be 7,334 ha, with 4,093 ha, 2,504 ha, and 737 ha in Washington, California, and Oregon, respectively (USDA-NASS, 2022). The total value of this crop was $531 million in 2021 (USDA-NASS, 2022). Red raspberry production faces many challenges that threaten the industry’s survival. Hot and dry weather has severely impacted raspberry quality and yield in California and Washington (USDA-NASS, 2022). Furthermore, the production lifetime of plantings has significantly declined over the last 10 years owing to the impact of soilborne pathogens (Gigot et al., 2013; Rudolph and DeVetter, 2015). Two of the most important soilborne pathogens limiting raspberry production in the PNW are *Phytophthora rubi* and *Pratylenchus penetrans* (Wilcox et al., 1993; Zasada et al., 2015; Weiland et al., 2018). Historically, management of these organisms was achieved through pre-plant broadcast fumigation (Walters et al., 2017). However, new regulations have made fumigation more difficult and expensive. Therefore, understanding the ecology, distribution, and impact of soilborne pathogens in commercial red raspberry fields is considered a top research priority for growers in the region.

*Phytophthora rubi* is a homothallic oomycete that causes root rot in raspberries (Wilcox et al., 1993). The pathogen is commonly found in the PNW and severely affects the growth and development of raspberry (Gigot et al., 2013; Weiland et al., 2018). Over 99% of the isolates obtained from raspberry fields were *P. rubi*, indicating that this pathogen is the predominant causal agent of root rot of raspberry in the region (Stewart et al., 2014; Weiland et al., 2018, 2024). Symptoms associated with *Phytophthora* root rot include root necrosis, leaf chlorosis, wilting, stunting, reduced cane and fruit production, and plant death. Warm soil temperatures ≥15°C favor pathogenesis, and wet soils enable the pathogen to form sporangia and release infective motile zoospores (Duncan et al., 1991; Graham et al., 2021).

*Pratylenchus penetrans* is a migratory endoparasitic nematode that invades and colonizes the root system (Kroese et al., 2016). Belowground symptoms often appear as necrotic spots or lesions while aboveground symptoms include chlorotic foliage and reduced growth. *P. penetrans* is widespread in fields where raspberry is grown (McElroy, 1977; Trudgill and Brown, 1978) and was detected in 100% of surveyed fields in Washington (Gigot et al., 2013). *P. penetrans* can negatively affect raspberry establishment and long-term productivity. In Washington, plant biomass and yield of raspberry were reduced by up to 92% when established in soil infested with *P. penetrans* compared to non-infested soil (Zasada et al., 2015).

Due, in part, to the presence of *P. rubi* and *P. penetrans*, the productive lifespan of raspberry fields in the PNW has decreased from over 10 years to 5–7 years (Wilcox et al., 1993). Currently, disease management revolves around using pre-plant fumigation and post-plant fungicide or nematicide treatments; however, both practices have limitations, and there is increasing evidence that adequate, long-term disease control has yet to be achieved (Rudolph and DeVetter, 2015; DeVetter et al., 2018; Weiland et al., 2018, 2024). Recent changes in regulations have made it increasingly difficult for growers to rely on soil fumigation as a management practice (US EPA, 2012). These new regulations have caused changes to allowable practices, including buffer zones, fumigation management plans, worker protection, and posting requirements. A concern regarding using post-plant fungicides is the development of fungicide resistance. In raspberries, the primary fungicides used are mefenoxam and phosphorous acid. However, there has been little to no evidence for resistance to these two fungicides in several surveys of *P. rubi* from the PNW region of North America, including British Columbia (Stewart et al., 2014; Sapkota et al., 2023; Weiland et al., 2024). Instead, evidence suggests that growers may be applying fungicides at the wrong time of year and perhaps to the wrong part of the plant to achieve adequate control (Weiland et al., 2024).

A successful disease management program requires detailed information on the spatial distribution and population dynamics of the pathogen. Spatial statistics have been widely used in plant pathology to understand the ecology and spread of pathogens in a cropping system (Turechek and Madden, 1999; Madden et al., 2007; Contina et al., 2018). Furthermore, spatial analysis provides additional insight for selecting effective sampling methods for pathogen detection in the field and identifying spatiotemporal biotic or abiotic risk factors driving the pathogenesis and disease epidemics (Fleischer et al., 1999; Gavassoni et al., 2001; Jaime-Garcia et al., 2001; Contina et al., 2020). Finally, these techniques provide a framework for implementing an integrated pest management strategy that minimizes the risk of severe crop losses and disease resistance development, maximizes the effectiveness of control measures, and increases growers’ return on investment.

Abiotic factors, such as soil moisture and temperature, have been widely found to influence disease incidence and severity levels (Yan and Nelson, 2022). Soil textural heterogeneity may lead to spatial heterogeneity in the growth and proliferation of soilborne pathogens (Curd et al., 2018). A survey of soilborne pathogens in raspberry found *P. rubi* negatively correlated with silt content and higher population densities of *P. penetrans* in soils with more clay (Gigot et al., 2013). The effect of soil textural heterogeneity will depend on the ability of soilborne pathogens to successfully migrate through the soil matrix, overcome the different competing trophic levels, and ultimately colonize the root infection court (Knudsen and Dandurand, 2014). Nematodes have been shown to depend on thin water films to swim through soil (Neher, 2010); bacteria have limited capacity for self-movement (Yang and van Elsas, 2018); and soilborne fungi spread predominantly through air-filled pore volume in permeable soils (Knudsen and Bin, 1990; Otten and Gilligan, 2006). However, agricultural machinery and equipment often mediate the long-distance dispersal of soil pathogens across the field. Disentangling the soil ecosystem complexities has proven to be a challenging and daunting task. Therefore, there is a need to use multivariate spatial statistical modeling to capture complex soil habitat interactions between biotic and abiotic factors.

Here, we investigated the spatial distribution of *P. rubi* and *P. penetrans* in commercial red raspberry fields in Oregon and Washington. Additionally, we investigated the influence of soil texture and elevation on the prevalence and distribution of *P. rubi* and *P. penetrans* in infested fields. Utilizing geostatistical tools, the objectives of this study were to (i) determine the spatial distribution of *P. rubi* and *P. penetrans* in four infested fields in Oregon and Washington, (ii) evaluate the influence of soil texture and elevation on the landscape distribution of *P. rubi* and *P. penetrans*, (iii) determine the effects of *P. rubi* and *P. penetrans* on disease severity levels in the field, and (iv) determine the effects of *P. rubi* and *P. penetrans* in soil on the level of *P. penetrans* infestation in roots.

## Materials and Methods

### Study location

Four commercial red raspberry fields were sampled for this study. One field was sampled in each Gresham, Oregon (OR-1) and Lynden, Washington (WA-1) in March 2014, and a second pair of fields was sampled in April 2015 in Portland, Oregon (OR-2) and Lynden, Washington (WA-2). The fields were chosen because they represented a diversity of raspberry growing environments in the region and also had areas that varied in plant vigor. All the fields were planted with the same raspberry variety, “Meeker”, and were naturally infested with *P. rubi* and *P. penetrans*. The ages of each field were OR-1 (23 years), OR-2 (12 years), WA-1 (5 years), and WA-2 (4 years). The soil type associated with each field was (USDA Soil Survey, 2024) OR-1 (Powell silt loam), OR-2 (Sauvie silt loam), WA-1 (Lynden sandy loam), and WA-2 (Whatcom silt loam).

### Field sampling and data collection

An area of approximately 4 ha that contained plants with and without symptoms of *Phytophthora* root rot was delineated in each field. Within this area, a 6 × 10 uniform grid of 60 sampling points was established, and root and soil samples were collected at each grid point (Miller et al., 1997). GPS coordinates were recorded at each sampling point using a GeoXT 2005 series GPS unit (Trimble, Sunnyvale, CA). The data collected are represented as a set of marked points illustrated in algebraic terms:

$$y=\left\{\left(x_{i},m_{i}\right),\ldots ,\left(x_{n},m_{n}\right)\right\}$$

Where *x_i_* is the sampling point locations (Easting and Northing), and *m_i_* is the attribute values (disease rating, *P. rubi* DNA in root, *P. penetrans* in root and soil, soil texture, and elevation) associated with each sampling point.

**Figure 1:**
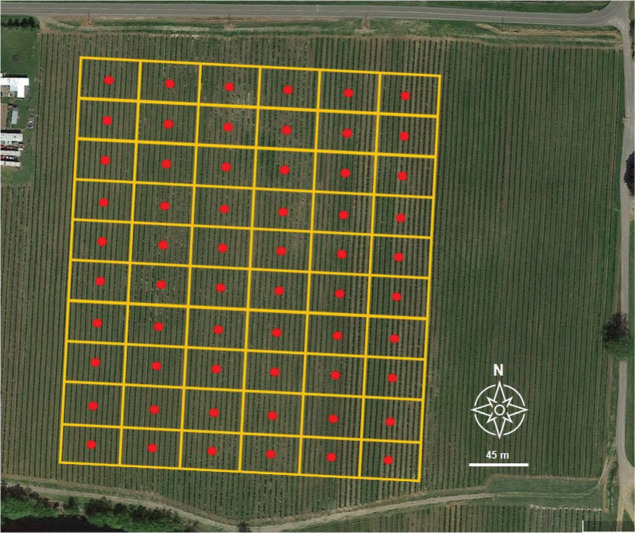
Layout schematic of a 6 × 10 grid sampling of contiguous quadrats (20 m × 45 m) in the commercial red raspberry production field WA-2 located in Lynden, WA. From the center of each quadrat (red dot), above-ground disease severity was visually assessed and rated; soil and root samples were collected to quantify *Phytophthora rubi*, *Pratylenchus penetrans*, and soil texture (sand, silt, clay); and surface field elevations were also recorded at each sampling point.

Due to differences in the field layout, the distance between each sampling point varied by field. Sampling points at OR-1 and OR-2 were 25 m × 35 m, while those at WA-1 and WA-2 were 35 m × 20 m and 45 m × 20 m, respectively (Fig. 1). Root samples were collected from a single plant at each sample site. Due to differences in plant health, not all samples had the same amount of collected root material, but generally, 5–10 g of root material was collected from each location. The next nearest plant was sampled if no plant was present at a designated sampling point. Approximately 500 g of soil from around the root system was collected using a shovel along with root material. Roots and soil from each sampling point were stored in the same bag and processed in the laboratory. Samples were passed over a 2-mm sieve to separate roots from the soil, and the soil sample was partitioned to facilitate different analyses.

### *P. penetrans* extraction

A 50-g subsample of soil was placed on a Baermann funnel to extract *P. penetrans* (Walters et al., 2017). Nematodes were collected after 5 days and enumerated using a dissecting microscope. A separate 50-g subsample of soil was placed in a 65°C oven for 5 days before being weighed to obtain soil moisture. Roots from each sample were rinsed with water to remove excess soil and then patted dry with a paper towel. Fine roots (<1 mm) were separated from larger roots, and approximately 1 g of fine roots was placed in a 20-ml glass scintillation vial and then stored at −80°C until further processing for *P. rubi*. The remaining fine roots were used for *P. penetrans* extractions, and larger roots were discarded. Fine root material was placed under intermittent mist for 5 days to extract *P. penetrans* (Walters et al., 2017). After nematode extraction, the root material was placed in a 65°C oven, and after 5 days, the dry weight was determined. *P. penetrans* were enumerated using a dissecting microscope.

### *P. rubi* DNA extraction and disease rating

Frozen fine root samples for *P. rubi* were processed by flash freezing in liquid nitrogen and then pulverized using a tissue pulverizer (Garcia Manufacturing, Visalia, CA) for 20 s. Pulverized roots were transferred into a 1.7 ml centrifuge tube and stored at −80°C until used for DNA extractions. DNA was then extracted from 100 mg of pulverized fine root tissue using a FastDNA SPIN Kit for Soil (MP Biomedicals, Santa Ana, CA) following the manufacturer’s instructions. Extracted DNA was then used to conduct a *Phytophthora* species-specific quantitative real-time polymerase chain reaction (qPCR) assay described by Bilodeau et al. (2014) with the following modifications. Assays were run on the StepOnePlus machine and software (Thermo Fisher Scientific, Waltham, MA) using PerfeCta Multiplex qPCR ToughMix (Quanta Biosciences, Gaithersburg, MD). Only probes for *P. rubi* and the plant internal positive control (IPC) were used. The target for the species-specific qPCR assay was the atp9-nad9 region of the mitochondrial genome. The amplification master mix contained the genus-specific primer pair at 0.5 μM and the species-specific *P. rubi* probe at 0.1 μM. The primers for the plant internal control were present at 0.0125 μM and the probe at 0.01 μM. The cycling conditions for the assay were set at 95°C for 2 min, 50 cycles at 95°C for 15 s, and 60°C annealing temperature for 1 min 30 s, in a reaction volume of 25 μl. Each qPCR run also contained a set of *P. rubi* DNA standards in the quantities of 2 ng, 0.2 ng, and 0.02 ng, and each standard or sample was run in triplicate (Bilodeau et al., 2014). Following the initial sampling of roots and soil in spring, each field was revisited in summer, and a visual disease rating assessment was performed for each plant at each sampling point. The ratings were based on a 1–10 scale, with 1 representing a healthy and vigorous plant with uniform growth, 5 representing a plant with moderate reduction in growth, and 10 representing an entirely dead plant (T. W. Walters, personal communication).

### Soil texture analysis

Soil texture was conducted by A & L Western Labs (Portland, OR). A subsample of soil from each sampling point was analyzed for sand, silt, and clay content.

### Spatial dependency and heterogeneity

Global Moran’s I was used to assess spatial autocorrelation in the distribution of disease rating, *P. rubi* DNA in root, *P. penetrans* in root and soil, soil texture, and elevation in the field (Rossi and Quénéhervé, 1998; Holguin et al., 2015). It was developed to test the null hypothesis of spatial independence or zero spatial autocorrelation (*P* > 0.05). The test statistic is a weighted product–moment correlation coefficient reflecting geographic proximity (Moran, 1950):

$$I=\frac{n}{S_{0}}\frac{\sum_{i=1}^{n}\sum_{j=1}^{n}w_{i,j}\left(y_{i}-\bar{y}\right)\left(y_{j}-\bar{y}\right)}{\sum_{i=1}^{n}{\left(y_{i}-\bar{y}\right)}^{2}}$$

Where *y_i_* are the observations, *w_i,j_* represents the distance weight (distance matrix), *n* is the number of observations, and $S_{0}=\sum_{i=1}^{n}\sum_{j=1}^{n}w_{i,j}$ is the sum of all *w_i,j_*.

Global Moran’s I coefficient values range from −1 to 1, where a value <0 indicates negative spatial autocorrelation and a value >0 indicates positive spatial autocorrelation. A positive spatial autocorrelation indicates the presence of clustering, i.e., nearby areas have similar attribute values, whereas a negative spatial autocorrelation indicates dissimilarities in neighborhood data attribute values.

Local Moran’s I, also known as local indicators of spatial association (LISA), was used to identify local spatial clusters and outliers in the distribution of disease rating, *P. rubi* DNA in root, *P. penetrans* in root and soil, soil texture, and elevation for each field under the assumption of spatial heterogeneity (Anselin, 1995). The local spatial statistic Moran’s I is calculated for each sampling point based on a predefined spatial weight and is expressed as:

$$I_{i}=\frac{\left(y_{i}-\bar{y}\right)}{\sum_{k=1}^{n}{\left(y_{k}-\bar{y}\right)}^{2}/\left(n-1\right)}\sum_{j=1}^{n}w_{ij}\left(y_{j}-\bar{y}\right)$$

Where a positive value for *I_i_* indicates that a sampling point has a similar attribute value to the neighboring points (clusters), and a negative value indicates dissimilar attribute values in the local neighborhood (outliers).

Variogram modeling was used to determine and quantify spatial variability of disease rating, *P. rubi* DNA in root, *P. penetrans* in root and soil, soil texture, and elevation (Larkin et al., 1995; Miller et al., 1997; Madden et al., 2007). A variogram is the expected squared difference between two data values separated by a distance vector (Cressie, 1993; Wackernagel, 2003). The semivariogram represents half the variogram and was first introduced by Matheron (1963).

$$\gamma \left(d\right)=\frac{1}{2}\sum \left\{{\left[z\left(x_{i}\right)-z\left(x_{j}\right)\right]}^{2}\right\}$$

Where *γ*(*d*) is the semivariance values as a function of lag distance and *z*(*x_ij_*) is the point locations associated with their attribute values.

Parameters associated with the variogram include nugget (the short-range variability in the data), sill (the total variance of the empirical variogram), and range (the distance after which the data are no longer correlated). Variogram points above the sill indicate negative spatial autocorrelation and points below the sill indicate positive spatial autocorrelation. The variogram is fitted with a specific known mathematical function such as the spherical, exponential, Gaussian, or Matern models.

The ordinary kriging was used as a probabilistic interpolator to predict disease rating, *P. rubi* DNA in root, *P. penetrans* in root and soil, soil texture, and elevation at one or more unsampled points. It is considered the best unbiased linear interpolation method, where the expectation of errors equals zero, and the predicted value is a linear combination of weighted known values (Cressie, 1990):

$$\hat{z}\left(x_{0}\right)=\sum_{i=1}^{n}\lambda_{i}z\left(x_{i}\right)$$

Where *λ*_*i*_ are the weights and *z*(*x_i_*) is the known value of the variable *z* at sampling point *x_i_*.

Kriging assumes spatial variation to be statistically consistent across the field and considers that an attribute value is neither completely random nor completely determined. Prediction is codetermined by spatial autocorrelation factors, offsets, and random error (Cressie, 1988, 1990).

### Classical and spatial regression modeling

Ordinary least squares (OLS) multiple regression is a classical statistical method routinely used to predict dependent variables measured at the interval or ratio level. The OLS multiple regression model is expressed as (Ott and Longnecker, 2016):

$$y=\beta_{0}+\beta_{1}X_{1}+\beta_{2}X_{2}+\ldots +\beta_{k}X_{k}+\varepsilon$$

Where *y* is the predicted value of the dependent variable, *X*_1_ through *X_k_* are *k* distinct independent variables, *β*_0_ is the intercept, the value of *y* when all the independent variables are equal to zero, and *β*_1_ through *β*_*k*_ are the estimated regression coefficients.

In OLS, the estimates and standard errors (SE) are unbiased if the required assumptions are fulfilled—linearity, homoscedasticity, independence of errors, normality, and independent variables (Ott and Longnecker, 2016; James et al., 2021). With spatial dependency, using OLS will produce erroneous predictions due to spatial autocorrelation in the predictor or the OLS regression model residuals, leading to biased parameter estimates (Lopez-Nicora et al., 2020). Therefore, spatial regression models are appropriate for accommodating spatial dependency in either the response variable or the regression model errors (Bivand et al., 2013). The spatial lag model (SLM), spatial Durbin model (SDM), spatial error model (SEM), conditional autoregressive (CAR) model, and spatial autoregressive moving average (SARMA) are widely used to account for spatial interactions between autocorrelated dependent and independent variables (Anselin, 1988; Madden et al., 1988; Bivand et al., 2013; Plant, 2019) and were therefore used in this study.

Spatial lag model, also known as the spatial autoregressive model, only considers the effect of the spatial lag on the dependent variable while focusing on the interaction among observations (Whittle, 1954; Anselin, 1988, 1992) and is defined as:

$$y=\beta_{0}+\rho w_{i}y_{j}+\beta_{1}X_{1}+\beta_{2}X_{2}+\ldots +\beta_{k}X_{k}+\varepsilon$$

Where *ρ* is the spatial coefficient and *w_i_y_i_* is the spatially lagged dependent variable for the weights matrix *w_i_*. The parameter *ρ* also specifies the degree of spatial dependence; when it is positive, a positive correlation exists; and when negative, a negative correlation exists. If there is no spatial dependence on the independent variables, *y* does not depend on *y_i_,* then *ρ* = 0, and the equation reverts to a classical OLS multiple regression.

Spatial Durbin model is a modification of SLM and considers the spatial lag’s effect on the independent and dependent variables (LeSage and Pace, 2009) and is defined as:

$$y=\beta_{0}+\rho w_{i}y_{j}+w_{1}X\beta_{2}+\beta_{1}X_{1}+\beta_{2}X_{2}+\ldots +\beta_{k}X_{k}+\varepsilon$$

Where *w_1_Xβ_2_* is the spatial lag effect of the independent variables.

Spatial error model considers the spatial lag’s effect in the error term and does not include lagged dependent or independent variables (Anselin, 1992). The error is split into random error and spatially structured error and is defined as:

$$y=\beta_{0}+\lambda w_{i}\varepsilon_{i}+\beta_{1}X_{1}+\beta_{2}X_{2}+\ldots +\beta_{k}X_{k}+\varepsilon$$

Where λ is the spatial error coefficient and *w_i_*ɛ_*i*_ is the spatially lagged error term for weights matrix *w_i_*. If there is no spatial correlation between the random and spatially structured errors, then λ = 0.

Conditional autoregressive focuses on the conditional distribution of the spatial error terms (Cliff and Ord, 1981; Schabenberger and Gotway, 2005; Bivand et al., 2013) and is defined as:

$$e_{i}|e_{j\sim i}\sim N\left(\sum_{j\sim i}\frac{c_{ij}e_{j}}{\sum_{j\sim i}c_{ij}},\frac{\sigma_{e_{i}}^{2}}{\sum_{j\sim i}c_{ij}}\right)$$

Where *e_i_* is the conditional distribution, *N* denotes a normal distribution with the mean and variance parameters, $\sigma_{e_{i}}^{2}$ is the conditional variance, and *c_i,j_* is the spatial dependence between point locations.

Spatial autoregressive moving average is used for modeling local autocorrelation and can account for spatial dependence among the error terms, like SEM, and the dependent variable, like SLM (LeSage and Pace, 2009), an d is defined as:

$$y=\beta_{0}+\rho w_{i}y_{j}+\lambda w_{i}\varepsilon_{i}+\beta_{1}X_{1}+\beta_{2}X_{2}+\ldots +\beta_{k}X_{k}+\varepsilon$$

### Data analysis and modeling

This study used the software R version 4.4.0 as a modeling language environment for geostatistical analysis and disease mapping (R Core Team, 2018). Exploratory data analysis was performed, and values of disease rating, *P. rubi* DNA in root, *P. penetrans* in root and soil, soil texture, and elevation were summarized for each field. *P. rubi* DNA values and *P. penetrans* in soil and root were normalized with Log_10_-transformation. Arcsine transformations were applied to disease rating, sand, silt, and clay, and elevation values were square root transformed. The data were transformed into a spatial data frame containing point locations and associated attribute values (disease rating, *P. rubi* DNA in root, *P. penetrans* in root and soil, soil texture, and elevation) using the function “SpatialPointsDataFrame” within the package “sp” (Pebesma and Bivand, 2005). Spatial matrices and weights were created based on nearest neighbors—with 15 links on average—using the functions “knearneigh,” “knn2nb,” and “nb2listw” from the package “spdep” (Bivand et al., 2024a, 2024b).

Global Moran’s I test for spatial autocorrelation was computed using the function “moran.test” within the package “spdep” (Bivand et al., 2024a, 2024b), and the Global Moran’s I coefficient and *P*-value were extracted and compiled in a summary table for each field. Local Moran’s I test for spatial association was computed using the function “localmoran” from the package “spdep” (Bivand et al., 2024a, 2024b). Variogram analysis was performed using the function “autofitVariogram.” The function is available within the package “automap” (Hiemstra and Skoien, 2023). Directional variogram analysis was performed using the function “variogram” from the package “gstat” to assess for anisotropy in the spatial distribution of disease rating, *P. rubi* DNA in root, *P. penetrans* in root and soil, soil texture, and elevation across the field (Pebesma and Graeler, 2023). The ordinary kriging was performed to map disease rating, *P. rubi* DNA in root, *P. penetrans* in root and soil, soil texture, and elevation using the function “autoKrige” in the package “automap” (Hiemstra and Skoien, 2023).

Ordinary least squares multiple regression was used to assess the influence of soil texture and elevation on the distribution of *P. rubi* DNA in root and *P. penetrans* in root and soil for each field using the function “lm.” Additionally, the effects of *P. rubi* DNA in root and *P. penetrans* in root and soil on disease severity rating and the relationship between *P. penetrans* in soil and in roots were assessed in regression modeling. Homoscedasticity was assessed using the residuals from the fitted models and plotted against the predicted values, and multicollinearity was assessed using the model’s variance inflation factor (VIF) (James et al., 2021). Moran’s I test for spatial autocorrelation in the residuals was performed from the estimated linear model using the function “lm.morantest” from the package “spdep” (Bivand et al., 2024a, 2024b). Finally, SLM and SDM were computed using the function “lagsarlm,” SEM with the function “errorsarlm,” and CAR and SARMA with the function “spautolm” within the package “spatialreg” (Bivand et al., 2024a, 2024b). Akaike information criterion (AIC) was used to compare OLS, SLM, SDM, SEM, CAR, and SARMA models and to determine the best model that fits the data for each field (James et al., 2021). Subsequently, the model with the lowest AIC value was considered the best.

## Results

### Exploratory data analysis

Disease severity was worse in OR-1 and WA-2 than in OR-2 and WA-1 (*P* < 0.05). The variance-to-mean ratio (VMR) values showed that the distribution of disease severity values was under-dispersed for all the fields. *P. rubi* DNA concentrations in roots varied significantly across all four fields (*P* < 0.05) (Table 1). WA-1 had the highest average *P. rubi* DNA concentrations in roots, followed by WA-2, OR-1, and OR-2, and VMR values were highest in WA-1 and WA-2, indicating the presence of considerable variations in the distribution of *P. rubi* DNA concentrations in roots (Table 1). *P. penetrans* densities in roots were significantly higher in WA-2 and OR-2 and were the lowest in WA-1 and OR-1 (*P* < 0.05), with VMR values indicating strong variations in their distributions for each field (Table 1). *P. penetrans* densities in soil were significantly higher in WA-2 and more dispersed compared with the other fields (*P* < 0.05) (Table 1). Soil texture varied greatly across all four fields; WA-1 had the highest content of sand, while OR-1 and WA-2 had the highest silt content, and OR-2 had the highest content of clay (Table 1). For field elevation, only OR-1 had a significant elevation level compared to the other fields (*P* < 0.05) (Table 1).

**Table 1:** Exploratory data analysis of disease rating, *Phytophthora rubi* DNA concentrations in roots, *Pratylenchus penetrans* densities in roots and soil, soil texture, and elevation in four commercial red raspberry production fields in Oregon (OR-1 and OR-2) and Washington (WA-1 and WA-2).

**Field**	**Variables**	**Mean**	**SE**	**Standard deviation**	**VIR^a^**
**OR-1**	Disease rating	6.53	0.18	1.37	0.29
	*P. rubi* DNA (ng/g root)	681.87	192.78	1,493.29	3,270.30
	*P. penetrans*/g root	16.78	4.80	37.16	82.29
	*P. penetrans*/g soil	50.38	10.62	82.25	134.27
	% sand	27.93	0.50	3.90	0.54
	% silt	51.45	0.45	3.50	0.24
	% clay	20.61	0.34	2.61	0.33
	Elevation (m)	6.43	0.38	2.92	1.33

**OR-2**	Disease rating	3.65	0.08	0.62	0.10
	*P. rubi* DNA (ng/g root)	91.75	64.48	499.49	2,719.20
	*P. penetrans*/g root	337.32	79.88	618.74	1,134.95
	*P. penetrans*/g soil	86.05	9.06	70.17	57.22
	% sand	27.93	0.89	6.87	1.69
	% silt	45.97	0.88	6.88	1.03
	% clay	26.53	0.44	3.44	0.45
	Elevation (m)	0.66	0.05	0.39	0.23

**WA-1**	Disease rating	3.26	0.23	1.79	0.98
	*P. rubi* DNA (ng/g root)	1,106.31	514.86	3,988.13	14,376.77
	*P. penetrans*/g root	16.16	5.96	46.19	132.06
	*P. penetrans*/g soil	26.77	4.89	37.91	53.67
	% sand	62.60	0.89	6.87	0.75
	% silt	27.27	0.72	5.57	1.14
	% clay	10.13	0.29	2.26	0.50
	Elevation (m)	1.74	0.13	1.00	0.58

**WA-2**	Disease rating	5.47	0.22	1.73	0.54
	*P. rubi* DNA (ng/g root)	817.32	440.91	3,415.25	14,270.84
	*P. penetrans*/g root	598.35	97.64	756.33	956.02
	*P. penetrans*/g soil	178.42	26.01	201.51	227.58
	% sand	34.01	1.01	7.85	1.81
	% silt	51.75	0.92	7.11	0.98
	% clay	14.25	0.41	3.18	0.71
	Elevation (m)	1.43	0.09	0.73	0.37

a When <1, the distribution is under-dispersed, and when >1, the distribution is over-dispersed.

SE, standard errors; VMR, variance-to-mean ratio.

### Spatial dependency and heterogeneity

Global Moran’s I test was used to assess spatial autocorrelation in the distribution of the measured variable (Table 2). Disease severity and *P. rubi* DNA concentrations in roots had significant positive spatial autocorrelations in OR-1, WA-1, and WA-2 (*P* < 0.05). *P. penetrans* densities in roots had significant positive spatial autocorrelation only for WA-2 (*P* < 0.05), while *P. penetrans* densities in soil had significant positive spatial autocorrelation for OR-2, WA-1, and WA-2 (*P* < 0.05). Soil texture was positively significant for all fields except for clay content in OR-2 (*P* < 0.05), and field elevations were positively significant for all fields (*P* < 0.05).

**Table 2:** Global Moran’s I for assessing spatial autocorrelation in disease rating, *Phytophthora rubi* DNA concentrations in roots, *Pratylenchus penetrans* densities in roots and soil, soil texture, and elevation in four commercial red raspberry production fields in Oregon (OR-1 and OR-2) and Washington (WA-1 and WA-2).

**Field**	**Variables**	**Moran’s I coefficient^a^**	***P*-value^b^**
**OR-1**	Disease rating	0.28	<0.001
	*P. rubi* DNA in root	0.07	0.001
	*P. penetrans* in root	0.04	0.08
	*P. penetrans in soil*	0.04	0.07
	Sand	0.11	<0.001
	Silt	0.10	0.002
	Clay	0.18	<0.001
	Elevation	0.75	<0.001

**OR-2**	Disease rating	0.01	0.22
	*P. rubi* DNA in root	−0.02	0.53
	*P. penetrans* in root	−0.01	0.40
	*P. penetrans in soil*	0.06	0.01
	Sand	0.07	0.009
	Silt	0.06	0.01
	Clay	0.02	0.20
	Elevation	0.10	0.002

**WA-1**	Disease rating	0.21	<0.001
	*P. rubi* DNA in root	0.08	0.003
	*P. penetrans* in root	0.004	0.29
	*P. penetrans in soil*	0.31	<0.001
	Sand	0.28	<0.001
	Silt	0.19	<0.001
	Clay	0.31	<0.001
	Elevation	0.59	<0.001

**WA-2**	Disease rating	0.51	<0.001
	*P. rubi* DNA in root	0.22	<0.001
	*P. penetrans* in root	0.09	0.003
	*P. penetrans in soil*	0.11	<0.001
	Sand	0.42	<0.001
	Silt	0.22	<0.001
	Clay	0.43	<0.001
	Elevation	0.53	<0.001

a Global Moran’s I coefficient values range from −1 to 1, where a value <0 indicates negative spatial autocorrelation and a value >0 indicates positive spatial autocorrelation. A positive spatial autocorrelation indicates the presence of clustering, i.e., nearby areas have similar attribute values, whereas a negative spatial autocorrelation indicates dissimilarities in neighborhood data attribute values.

b The *P*-values indicate the level of significance at *P* < 0.05 for each coefficient.

**Figure 2:**
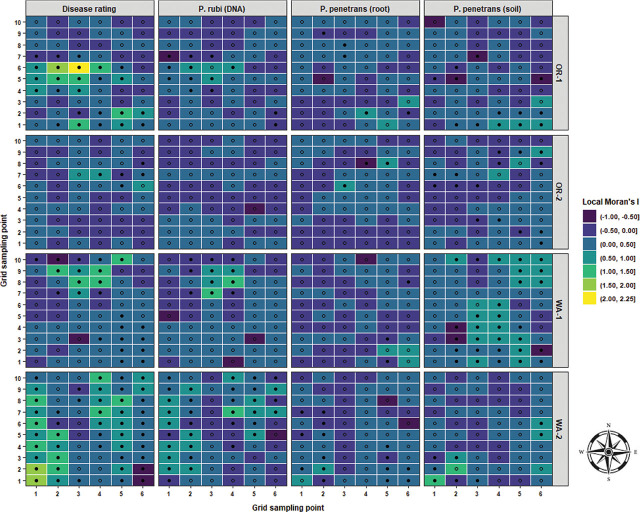
LISA for disease rating, *Phytophthora rubi* DNA concentrations in roots, and *Pratylenchus penetrans* densities in roots and soil in four commercial red raspberry production fields in Oregon (OR-1 and OR-2) and Washington (WA-1 and WA-2). A positive value for the local Moran’s I indicates that a sampling point has a similar attribute value to the neighboring points (clusters), and a negative value indicates dissimilar attribute values in the local neighborhood (outliers). Solid-filled black shapes indicate the significance of the local Moran’s I coefficient at *P* < 0.05. LISA, local indicators of spatial association.

**Figure 3:**
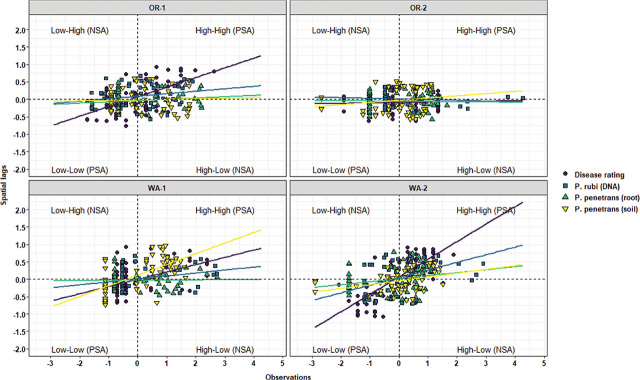
Moran scatterplot for disease rating, *Phytophthora rubi* DNA concentrations in roots, and *Pratylenchus penetrans* densities in roots and soil in four commercial red raspberry production fields in Oregon (OR-1 and OR-2) and Washington (WA-1 and WA-2). The scatterplot is characterized by four quadrants corresponding to the four types of spatial association and illustrates relationships between an individual sampling value (standardized observation) and the average values at neighboring sampling points (standardized spatial lags). The lower left and upper right quadrants indicate spatial clustering or PSA. The lower left quadrant is characterized by low values for both the spatial lags and the observations (Low–Low), and in the upper right quadrant, there are high values for both the spatial lags and the observations (High–High). The upper left (Low–High) and lower right (High–Low) quadrants indicate spatial outliers or NSA. The slope of each regression line corresponds to the Moran’s I coefficients listed in Table 2. NSA, negative spatial association; PSA, positive spatial association.

**Figure 4:**
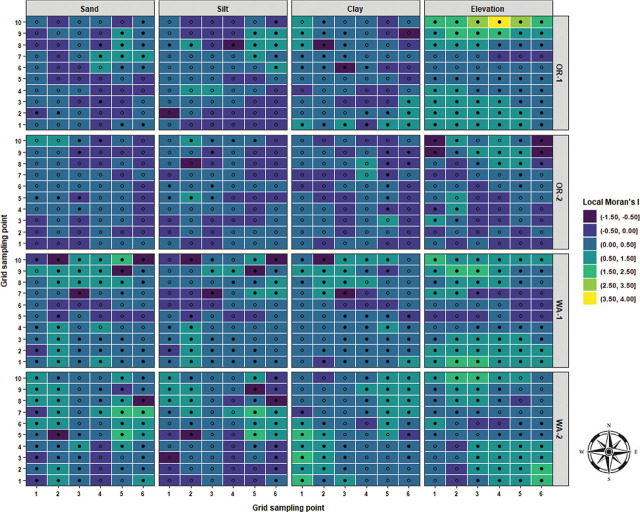
LISA for soil texture (sand, silt, and clay) and field elevation in four commercial red raspberry production fields in Oregon (OR-1 and OR-2) and Washington (WA-1 and WA-2). A positive value for the local Moran’s I indicates that a sampling point has a similar attribute value to the neighboring points (clusters), and a negative value indicates dissimilar attribute values in the local neighborhood (outliers). Solid-filled black shapes indicate the significance of the local Moran’s I coefficient at *P* < 0.05. LISA, local indicators of spatial association.

**Figure 5:**
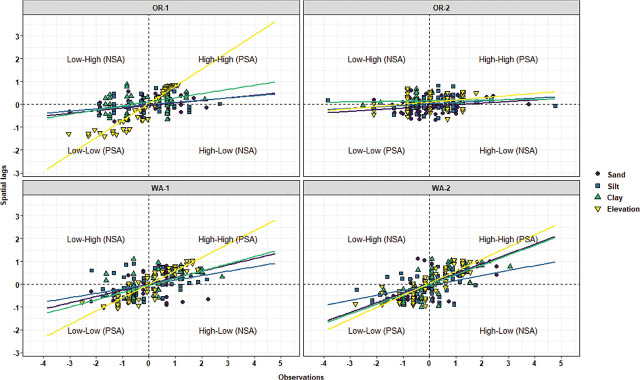
Moran scatterplot for soil texture (sand, silt, and clay) and field elevation in four commercial red raspberry production fields in Oregon (OR-1 and OR-2) and Washington (WA-1 and WA-2). The scatterplot is characterized by four quadrants corresponding to the four types of spatial association and illustrates relationships between an individual sampling value (standardized observation) and the average values at neighboring sampling points (standardized spatial lags). The lower left and upper right quadrants indicate spatial clustering or PSA. The lower left quadrant is characterized by low values for both the spatial lags and the observations (Low–Low), and in the upper right quadrant, there are high values for both the spatial lags and the observations (High–High). The upper left (Low–High) and lower right (High–Low) quadrants indicate spatial outliers or NSA. The slope of each regression line corresponds to the Moran’s I coefficients listed in Table 2. NSA, negative spatial association; PSA, positive spatial association.

Local Moran’s I test was performed to identify relationships and spatial associations between each observation of the measured variable with their surrounding neighbors. There were significant similarities or positive spatial associations (PSA) to neighboring sampling points had in 37% of the sampled grids for OR-1, 5% for OR-2, 30% for WA-1, and 72% for WA-2 for disease severity (*P* < 0.05) (Fig. 2). The PSA was significant in 12% of the sampled grids for OR-1, 0% for OR-2, 8% for WA-1, and 45% for WA-2 for *P. rubi* DNA in roots (*P* < 0.05) (Fig. 2). For *P. penetrans* in roots, the PSA was significant in 5% of the sampled grids for OR-1, 3% for OR-2, 0% for WA-1, and 15% for WA-2 (*P* < 0.05) (Fig. 2). The PSA was significant for *P. penetrans* in soil in 14% of the sampled grids for OR-1, 12% for OR-2, 44% for WA-1, and 10% for WA-2 (*P* < 0.05) (Fig. 2). The Moran scatterplot showed positive slopes for the regression lines for disease severity in all the fields; *P. rubi* DNA in roots in OR-1, WA-1, and WA-2; *P. penetrans* in roots in OR-1, WA-1, and WA-2; and *P. penetrans* in soil in all the fields (Fig. 3). The PSA was significant for soil texture, sand contents in 22% of the sampled grids for OR-1, 7% for OR-2, 52% for WA-1, and 67% for WA-2 (*P* < 0.05) (Fig. 4). Silt contents had significant PSA in 15% of the sampled grids for OR-1 and OR-2, 43% for WA-1, and 32% for WA-2 (*P* < 0.05) (Fig. 4). The PSA was significant for clay content in 31% of the sampled grids for OR-1, 3% for OR-2, and 53% for WA-1 and WA-2 (*P* < 0.05) (Fig. 4). Field elevation had significant PSA in 80% of the sampled grids for OR-1, 24% for OR-2, 68% for WA-1, and 63% for WA-2 (*P* < 0.05) (Fig. 4). There were positive slopes for the regression lines for soil texture and field elevation across all the fields according to the Moran scatterplot (Fig. 5).

**Figure 6:**
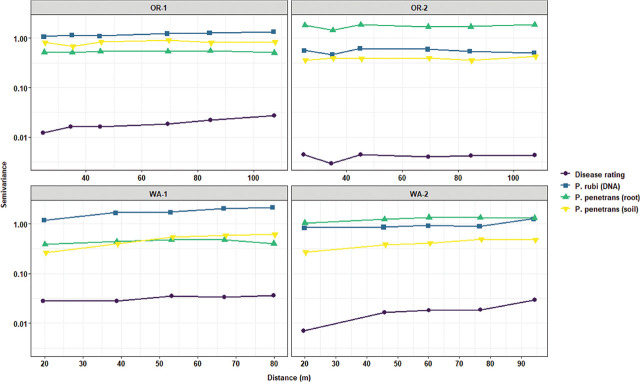
Experimental variogram graph for assessing the spatial structure of disease rating, *Phytophthora rubi* DNA concentrations in roots, and *Pratylenchus penetrans* densities in roots and soil in four commercial red raspberry production fields in Oregon (OR-1 and OR-2) and Washington (WA-1 and WA-2). The *x*-axis shows the distance lag, which is created by plotting the average variability between pairs of sampling points, and the *y*-axis shows the calculated value of the semivariance, where a greater value indicates less spatial correlation between pairs of points. The variogram model type and fitting parameters for each commercial field are listed in Table 3.

**Figure 7:**
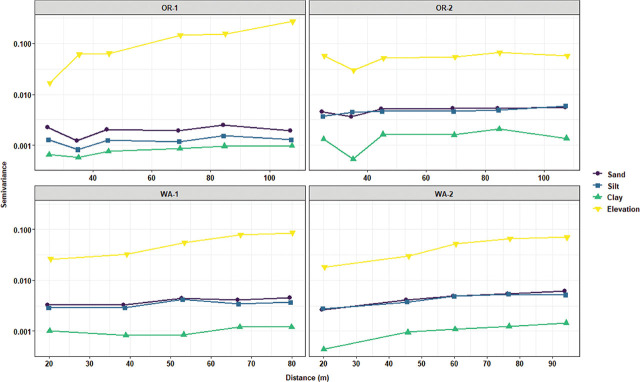
Experimental variogram graph for assessing the spatial structure of soil texture (sand, silt, and clay) and field elevation in four commercial red raspberry production fields in Oregon (OR-1 and OR-2) and Washington (WA-1 and WA-2). The *x*-axis shows the distance lag, which is created by plotting the average variability between pairs of sampling points, and the *y*-axis shows the calculated value of the semivariance, where a greater value indicates less spatial correlation between pairs of points. The variogram model type and fitting parameters for each commercial field are listed in Table 3.

Experimental variogram analyses were performed to evaluate the spatial variability and fit a spatial model to the distribution of the measured variable. Disease severity displayed some increased levels in the spatial continuity when plotting the distance lags against the semivariance for OR-1 and WA-2 (Fig. 6), and Gaussian and exponential models were fitted to their respective variograms (Table 3). *P. rubi* DNA concentrations and *P. penetrans* densities in roots were mostly characterized by steady and constant trend lines when assessing the semivariance over lag distances of pairwise points. *P. rubi* spatial continuity was more pronounced in WA-1, and *P. penetrans* in roots was highly defined in WA-2 (Fig. 6), and both were fitted with an exponential model (Table 3). *P. penetrans* densities in soil had a defined spatial continuity in WA-1 and WA-2 (Fig. 6); spherical and exponential models were fitted to their respective variograms (Table 3). There was strong spatial dependency for sand and silt contents in WA-1 and WA-2, for clay content in OR-2, and for field elevation in OR-1, WA-1, and WA-2 (Fig. 7).

**Table 3:** Fitted models for the experimental variogram analysis for quantifying the extent of spatial autocorrelation in disease rating, *Phytophthora rubi* DNA concentrations in roots, *Pratylenchus penetrans* densities in roots and soil, soil texture, and elevation in four commercial red raspberry production fields in Oregon (OR-1 and OR-2) and Washington (WA-1 and WA-2).

**Field**	**Variables**	**Model^a^**	**Nugget^b^**	**Sill^c^**	**Range^d^**
**OR-1**	Disease rating	Gaussian	0.01	0.02	102.27
	*P. rubi* DNA in root	Gaussian	1.05	0.44	100.72
	*P. penetrans* in root	Gaussian	0.50	0.03	29.52
	*P. penetrans* in soil	Gaussian	0.76	0.11	50.01
	Sand	Exponential	0.002	0.003	625.91
	Silt	Exponential	0.001	0.003	983.76
	Clay	Gaussian	0.001	0.0005	73.70
	Elevation	Gaussian	0.002	0.41	107.19

**OR-2**	Disease rating	Exponential	0.004	0.004	644.08
	*P. rubi* DNA in root	Gaussian	0.54	0.03	36.47
	*P. penetrans* in root	Exponential	1.62	1.17	674.35
	*P. penetrans* in soil	Exponential	0	0.39	10.03
	Sand	Gaussian	0.004	0.002	72.89
	Silt	Exponential	0.003	0.004	105.97
	Clay	Gaussian	0.001	0.0007	56.90
	Elevation	Exponential	0.044	0.15	861.29

**WA-1**	Disease rating	Gaussian	0.03	0.32	439.20
	*P. rubi* DNA in root	Exponential	0.37	1.98	37.66
	*P. penetrans* in root	Spherical	0.31	0.14	48.26
	*P. penetrans* in soil	Spherical	0.08	0.56	94.31
	Sand	Spherical	0.003	0.004	505.74
	Silt	Gaussian	0.003	0.002	75.60
	Clay	Exponential	0.001	0.0004	145.25
	Elevation	Gaussian	0.024	104.36	2,855.12

**WA-2**	Disease rating	Exponential	0.0005	0.09	265.02
	*P. rubi* DNA in root	Exponential	0.89	0.89	362.76
	*P. penetrans* in root	Exponential	0.62	0.74	23.00
	*P. penetrans* in soil	Exponential	0.13	0.42	49.90
	Sand	Gaussian	0.002	0.004	59.61
	Silt	Exponential	0.002	0.006	82.70
	Clay	Exponential	0	0.002	79.82
	Elevation	Gaussian	0.01	0.10	96.70

a The fitted mathematical model for the experimental variogram.

b The short-range variability in the data.

c The total variance of the empirical variogram.

d The distance after which the data are no longer correlated.

**Figure 8:**
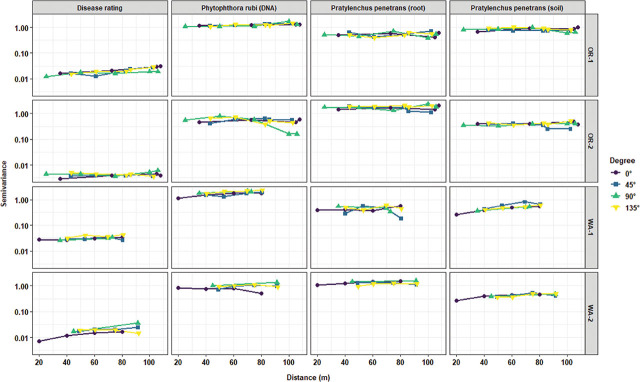
Directional experimental variogram graph for assessing potential directional variations in the spatial structure of disease rating, *Phytophthora rubi* DNA concentrations in roots, and *Pratylenchus penetrans* densities in roots and soil in four commercial red raspberry production fields in Oregon (OR-1 and OR-2) and Washington (WA-1 and WA-2). Directional angles used for the graph include 0° (North–South), 45° (Northeast–Southwest), 90° (East–West), and 135° (Southeast–Northwest). The overlapping of all the angle point lines indicates no directional variations in the spatial structure of the attribute values (isotropy), while major gaps between the angle point lines indicate the presence of directional trends (anisotropy).

**Figure 9:**
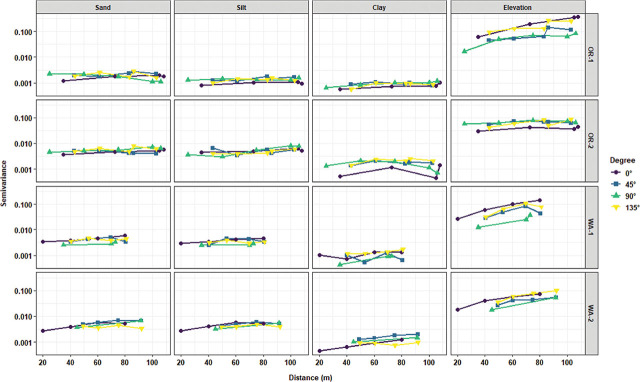
Directional experimental variogram graph for assessing potential directional variations in the spatial structure of soil texture (sand, silt, and clay) and field elevation in four commercial red raspberry production fields in Oregon (OR-1 and OR-2) and Washington (WA-1 and WA-2). Directional angles used for the graph include 0° (North–South), 45° (Northeast–Southwest), 90° (East–West), and 135° (Southeast–Northwest). The overlapping of all the angle point lines indicates no directional variations in the spatial structure of the attribute values (isotropy), while major gaps between the angle point lines indicate the presence of directional trends (anisotropy).

Directional variogram analyses were performed to test for the potential presence of anisotropy in the distribution of the measured variable. Overall, there were no major directional variations in the distribution of disease severity, *P. penetrans* densities in soil, and sand and silt contents. However, the presence of moderate anisotropy in the distribution of *P. rubi* DNA in roots in OR-2 and WA-2 and with *P. penetrans* densities in roots for WA-1 was detected (Fig. 8). There was moderate anisotropy for clay content in OR-2, WA-1, and WA-2. Field elevation had high levels of anisotropy for all the locations (Fig. 9).

### Spatial prediction and disease mapping

**Figure 10:**
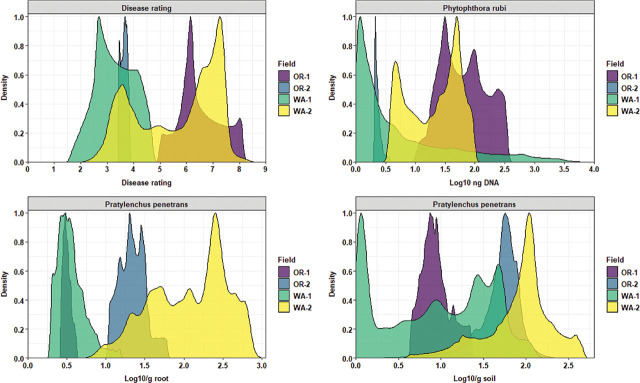
Kriging density graph for comparing the distribution of interpolated values of disease rating, *Phytophthora rubi* DNA concentrations in roots, and *Pratylenchus penetrans* densities in roots and soil across four commercial red raspberry production fields in Oregon (OR-1 and OR-2) and Washington (WA-1 and WA-2). The *x*-axis shows the attribute values of the variable, and the *y*-axis shows the proportion value assigned to each range of the attribute values.

Ordinary kriging was used as a probabilistic interpolator to estimate the values of the measured variable at unsampled locations in the fields. Before mapping the kriging predictions, density plots were used to capture and compare the distribution of the predictive outcomes. Disease severity values were skewed toward high disease levels for OR-1, restricted between lower and middle values for OR-2 and WA-1, and a bimodal distribution of two distinct peaks of low and high disease values for WA-2 (Fig. 10). *P. rubi* DNA concentrations in roots had a trimodal distribution centered on the average DNA values for OR-1; a single distribution peak of low DNA values for OR-2; a prominent positive skewness ranging from low to high DNA values for WA-1; and finally, a bimodal distribution of DNA values for WA-2 (Fig. 10). *P. penetrans* density in roots were restricted to low values dominated by a single distributional and overlapping peak for OR-1 and WA-1; a trimodal distribution centered slightly above the average values of nematodes in roots was recorded for OR-2; and WA-2 was dominated by a major and multiple minor peaks of high nematode values in roots (Fig. 10). *P. penetrans* densities in soil were dominated by a single major peak of distribution for OR-1 and OR-2, where OR-1 encompassed lower values while OR-2 higher values of nematodes in soil; an extensive multimodal distribution and skewed to lower values of nematodes in soil for WA-1; and finally, WA-2 was dominated by a single peak of high distribution values of nematodes in soil (Fig. 10).

**Figure 11:**
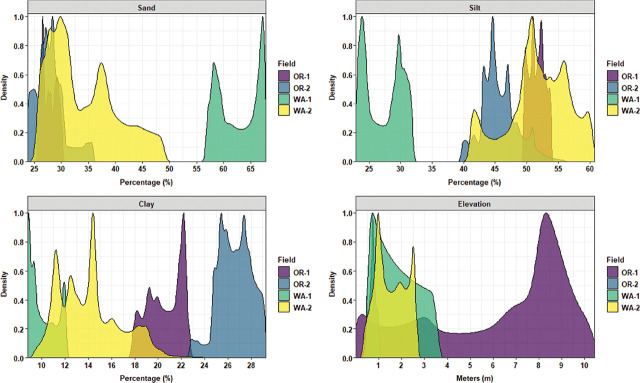
Kriging density graph for comparing the distribution of interpolated values of soil texture (sand, silt, and clay) and field elevation across four commercial red raspberry production fields in Oregon (OR-1 and OR-2) and Washington (WA-1 and WA-2). The *x*-axis shows the attribute values of the variable, and the *y*-axis shows the proportion value assigned to each range of the attribute values.

Sand content was characterized by low distribution values for OR-1, ranging between 25% and 30%, and OR-2, between 23% and 35%; WA-1 had the highest range of sand content (>55%) with a bimodal distribution; and finally, WA-2 encompassed a bimodal distribution centered to lower range values and an extended skewness reaching 50% of sand content (Fig. 11). Silt content was characterized by a cluster of multimodal density distributions in the upper level of high values for OR-1, OR-2, and WA-2, and WA-1 had the lowest range of values for silt content with a prominent bimodal distribution (Fig. 11). Clay content was characterized by a clear segmentation of OR-1 and OR-2, where OR-2 had the highest distribution levels of clay content; WA-1 dominated the lower range values for clay content; and WA-2 had a trimodal distribution and was skewed toward high values of clay content (Fig. 11). Field elevation was characterized by a cluster of low distribution values for OR-2, WA-1, and WA-2, and OR-1 had the highest distribution levels of elevation values with an extensive skewness toward low values (Fig. 11).

**Figure 12:**
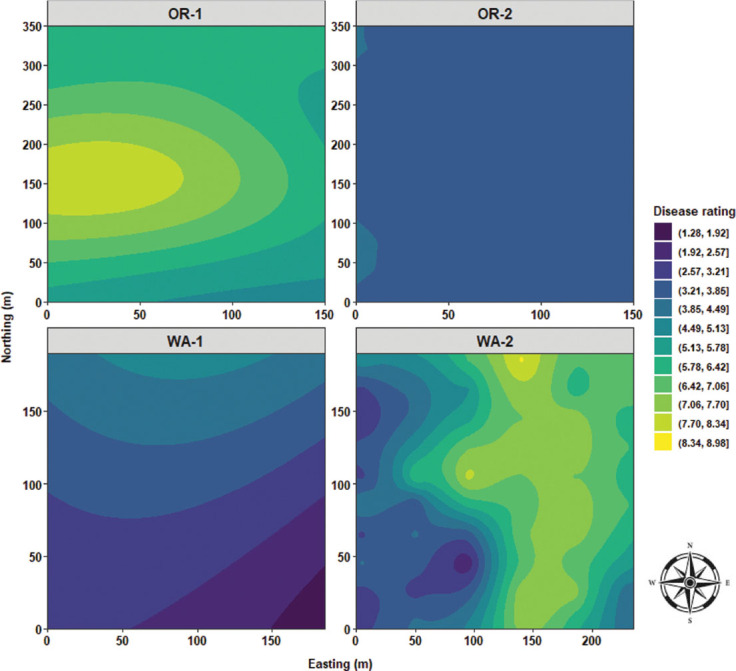
Spatial distribution of disease rating across four commercial red raspberry production fields in Oregon (OR-1 and OR-2) and Washington (WA-1 and WA-2). The ordinary kriging was used as a probabilistic interpolator to predict disease ratings at one or more unsampled points. Northing divides the map from north to south and Easting from west to east. Northing and Easting are grid references and are expressed in meters.

**Figure 13:**
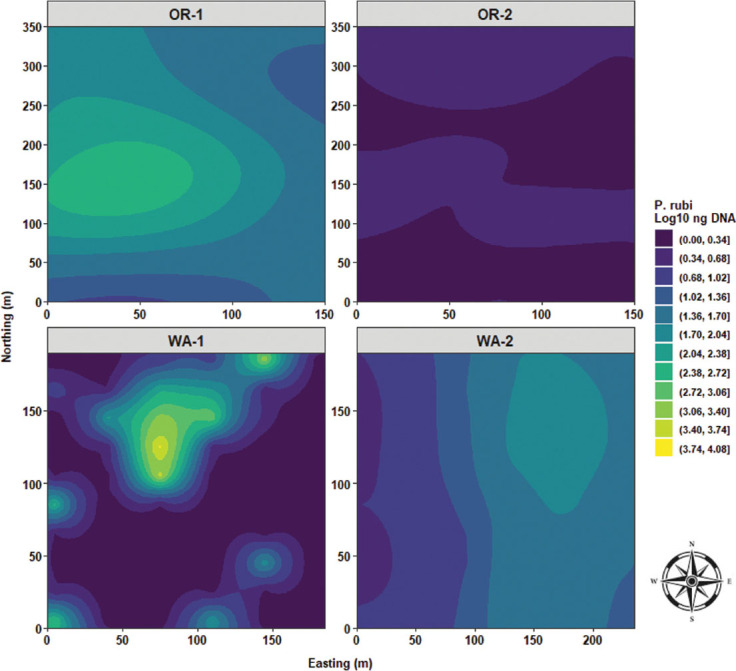
Spatial distribution of *Phytophthora rubi* DNA concentrations in roots across four commercial red raspberry production fields in Oregon (OR-1 and OR-2) and Washington (WA-1 and WA-2). The ordinary kriging was used as a probabilistic interpolator to predict *P. rubi* DNA in roots at one or more unsampled points. Northing divides the map from north to south and Easting from west to east. Northing and Easting are grid references and are expressed in meters.

Predictive maps generated by ordinary kriging analysis highlighted the distribution of the measured variables across the fields. Disease severity distribution was characterized by a single focus located westside of OR-1 with a concentric distributional ripple effect expanding eastbound. In OR-2 a monotonous distribution of disease severity values was observed. WA-1 had a descending wave of disease severity values from north to south. WA-2 was characterized by an elongated clump of high disease severity values, stretching from northeast to southeast with radiating waves toward both west and east sides of the field (Fig. 12). *P. rubi* DNA concentrations in roots followed similar distribution patterns to that of disease severity for OR-1 with overlapping ripple effect expanding eastbound. In OR-2 a quasi-monotonous distribution of low fungal DNA values in roots was observed. WA-1 was characterized by a major focus of high values of fungal DNA in roots and was located slightly toward the northwest side of the field and surrounded by several satellite clusters located toward the southeast, southwest, and west corner of WA-1. In WA-2, quasi-similar distribution patterns similar to disease severity were observed with an elongated focus of high fungal DNA values, stretching from the northeast to the southeast sides of WA-2 (Fig. 13).

**Figure 14:**
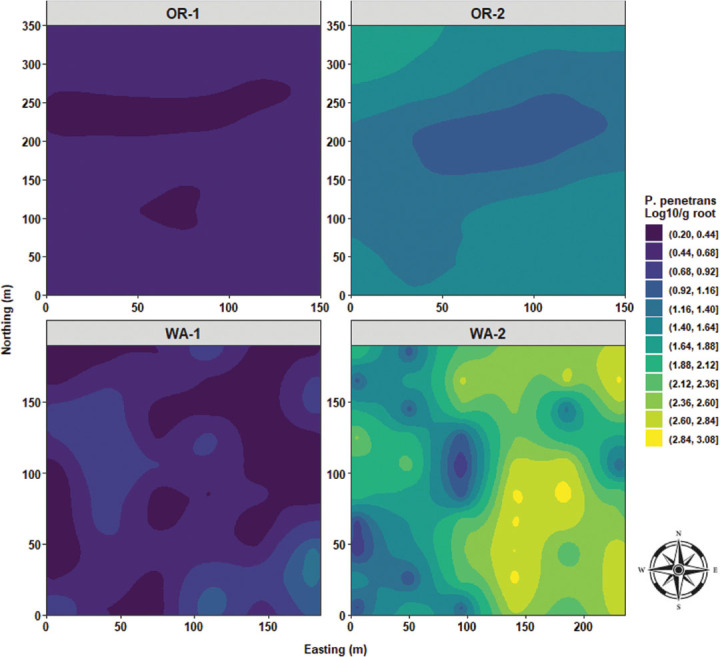
Spatial distribution of *Pratylenchus penetrans* densities in roots across four commercial red raspberry production fields in Oregon (OR-1 and OR-2) and Washington (WA-1 and WA-2). The ordinary kriging was used as a probabilistic interpolator to predict *P. penetrans* densities in roots at one or more unsampled points. Northing divides the map from north to south and Easting from west to east. Northing and Easting are grid references and are expressed in meters.

**Figure 15:**
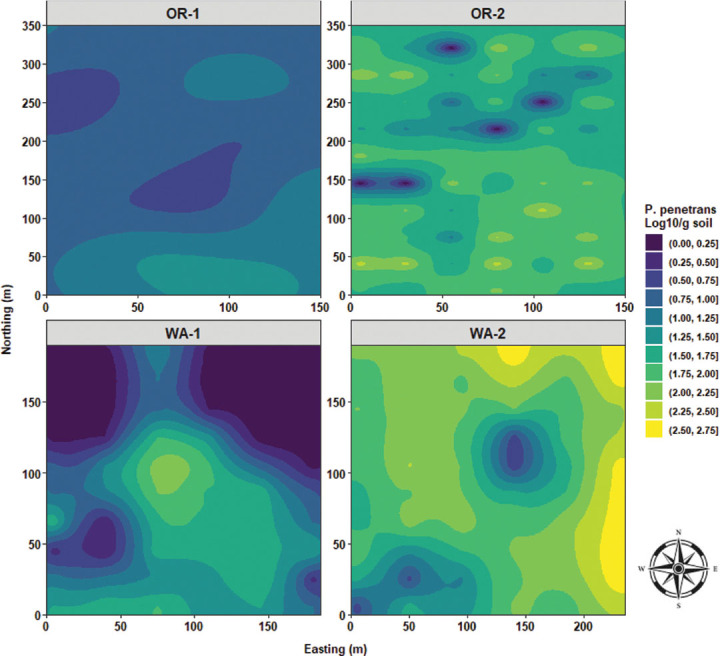
Spatial distribution of *Pratylenchus penetrans* densities in soil across four commercial red raspberry production fields in Oregon (OR-1 and OR-2) and Washington (WA-1 and WA-2). The ordinary kriging was used as a probabilistic interpolator to predict *P. penetrans* in the soil at one or more unsampled points. Northing divides the map from north to south and Easting from west to east. Northing and Easting are grid references and are expressed in meters.

In OR-1, *P. penetrans* densities in roots had a quasi-monotonous distribution of low nematode values with an elongated focus of low densities stretching from west to east. OR-2 is dominated by a large, centered clump of low *P. penetrans* densities in roots. WA-1 were characterized by a disorganized and nonstructured distribution pattern of low and high nematode densities in roots. In WA-2, quasi-similar distribution patterns similar to disease severity and *P. rubi* DNA concenrtrations in roots were observed, where high *P. penetrans* densities in roots were mostly found stretching from the northeast to the southeast sides of WA-2 (Fig. 14). In OR-1 and OR-2, *P. penetrans* in soil had the presence of multiple foci with low to median densities. WA-1 was characterized by a single clump of high nematode densities in soil with a rippling effect in all directions; however, large areas of extremely low nematode densities predominated northwest and northeast sides of WA-1. WA-2 was characterized by high *P. penetrans* densities in soil and predominantly circumscribed at the northeast, east, and southeast edges of the field, and a defined rippling wave of low *P. penetrans* densities was located slightly toward the center of WA-2 (Fig. 15).

**Figure 16:**
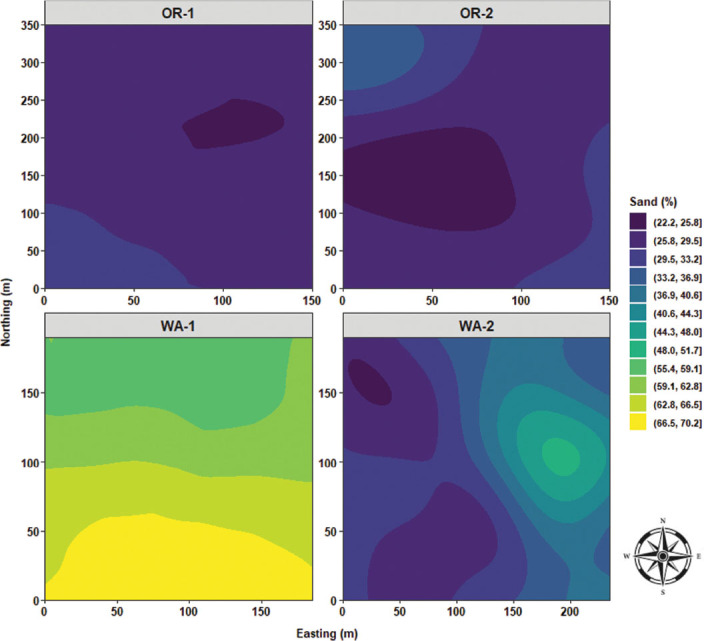
Spatial distribution of the percentage of sand across four commercial red raspberry production fields in Oregon (OR-1 and OR-2) and Washington (WA-1 and WA-2). The ordinary kriging was used as a probabilistic interpolator to predict the percentage of sand at one or more unsampled points. Northing divides the map from north to south and Easting from west to east. Northing and Easting are grid references and are expressed in meters.

**Figure 17:**
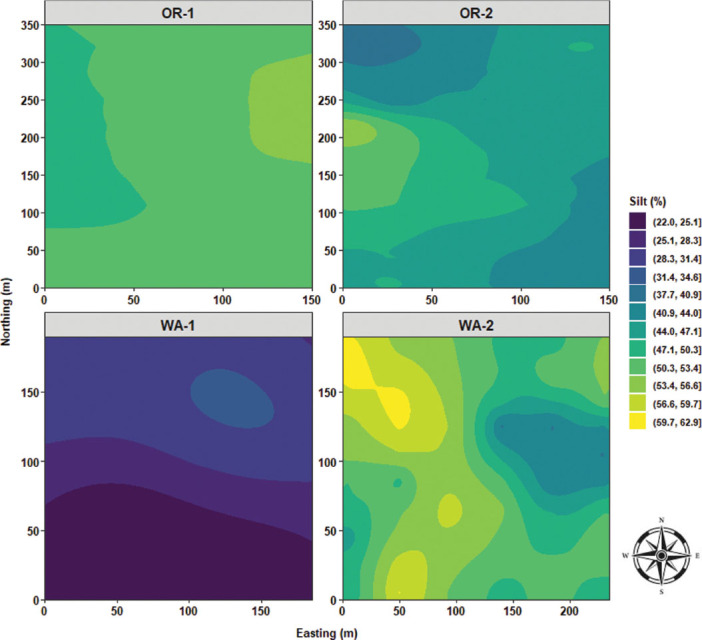
Spatial distribution of the percentage of silt across four commercial red raspberry production fields in Oregon (OR-1 and OR-2) and Washington (WA-1 and WA-2). The ordinary kriging was used as a probabilistic interpolator to predict the percentage of silt at one or more unsampled points. Northing divides the map from north to south and Easting from west to east. Northing and Easting are grid references and are expressed in meters.

**Figure 18:**
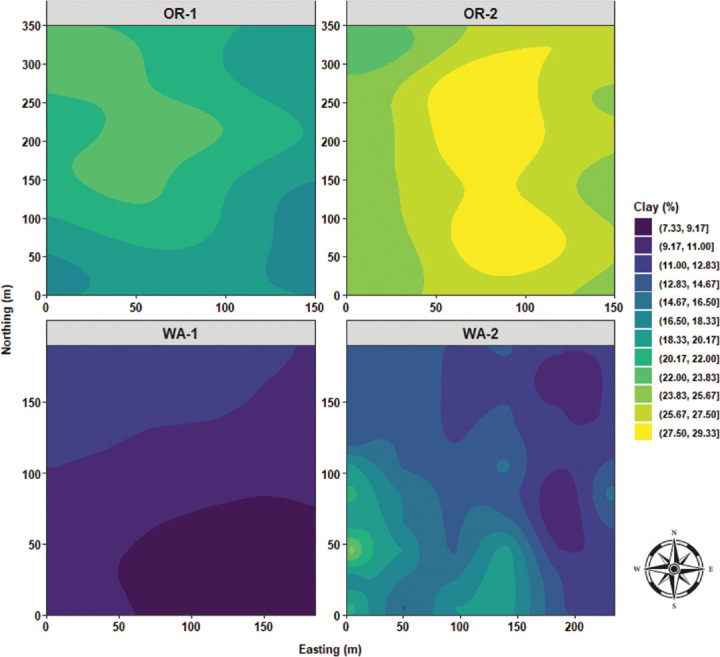
Spatial distribution of the percentage of clay across four commercial red raspberry production fields in Oregon (OR-1 and OR-2) and Washington (WA-1 and WA-2). The ordinary kriging was used as a probabilistic interpolator to predict the percentage of clay at one or more unsampled points. Northing divides the map from north to south and Easting from west to east. Northing and Easting are grid references and are expressed in meters.

Sand content had a quasi-monotonous distribution with a limited focus of low sand values for OR-1. OR-2 was characterized by a large clump of low sand values stretching from the westside to the center of the field, and median high values of sand were observed toward the northwest and southeast sides of OR-2. In WA-1, there was a massive area of extremely high sand content located southside of the field and radiating northward. In WA-2, quasi-similar distribution patterns of sand content similar to that of disease severity, *P. rubi* DNA concentration in roots, and *P. penetrans* densities in roots were observed with a stretching area of high sand content from northeast to southeast sides of WA-2 (Fig. 16). Silt content was predominated by a vast area amounting to over 50% of silt distribution for OR-1. OR-2 had multiple large swaths of areas encompassing silt content values ranging between 40% and 50%. WA-1 was characterized by mostly low values for silt content (22%–28%) located mostly in the southside of WA-1; however, a single clump of high silt content was observed in the northeast corner of the field. In WA-2, there were multiple foci of high silt content located northwest and southwest sides of the field (Fig. 17). Clay content was characterized by a large focus of high clay values located northwest corner of OR-1 and radiating southward. OR-2 had the highest level of clay content compared to the other fields and was characterized by a large centered clump spanning from north to south. WA-1 had a three-layered area encompassing low and median values for clay content. In WA-2, there was a focus of low values in the east and northeast sides and median values in the west and southwest corners of the field (Fig. 18).

**Figure 19:**
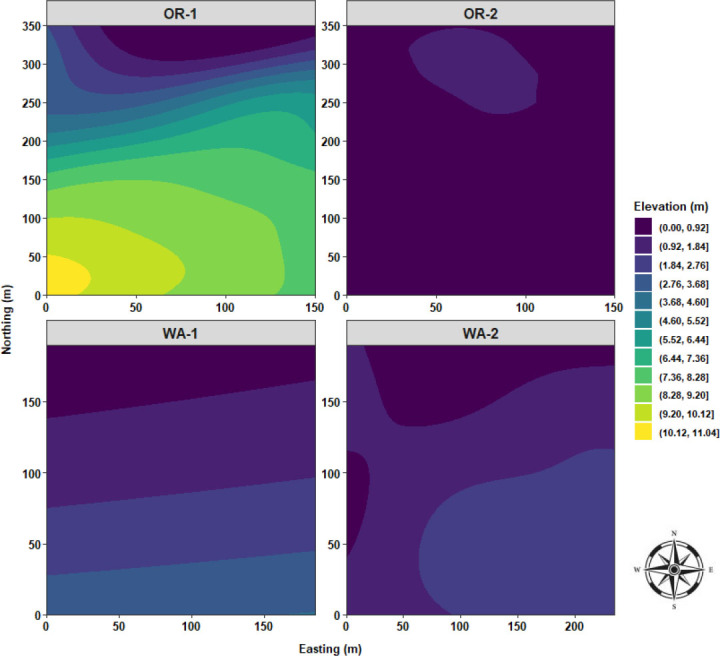
Spatial distribution of field elevation across four commercial red raspberry production fields in Oregon (OR-1 and OR-2) and Washington (WA-1 and WA-2). The ordinary kriging was used as a probabilistic interpolator to predict field elevation at one or more unsampled points. Northing divides the map from north to south and Easting from west to east. Northing and Easting are grid references and are expressed in meters.

Field elevation was predominated by a focus of high elevation values located southwest corner of OR-1 and radiating northeastward. In OR-2, there was a quasi-monotonous elevation distribution that was characterized by a single focus located north side of the field. WA-1 was represented by a four-layered area spanning from north to south with increasing elevation values. WA-2 followed similar patterns with a three-layered area spanning from northwest to southeast with increasing elevation values (Fig. 19).

### Effects of soil texture and field elevation on *P. rubi* and *P. penetrans* distributions

Ordinary least squares multivariate regression analyses were performed for each field location before performing Moran’s I test to detect spatial autocorrelation in the residuals. *P. rubi* DNA concentrations in roots was not significantly influenced by sand, silt, clay, and elevation for OR-1, OR-2, and WA-2 (*P* > 0.05), and a severe multicollinearity effect was observed for WA-1 due to overinflated variance (Table 4). Spatial autocorrelation in the residuals was significant only for WA-2 (*P* < 0.05) (Table S1 in Supplementary Materials). Based on a comparative regression model analysis, the SARMA model was able to detect significant effects of sand, silt, and elevation on *P. rubi* DNA concentrations in roots for OR-1 (*P* < 0.05; AIC = 179.81, *R*^2^ = 0.27). Additionally, the SARMA model revealed a significant influence of sand, silt, clay, and elevation on *P. rubi* DNA concentrations in roots for WA-1 (*P* < 0.05; AIC = 192.91, *R*^2^ = 0.33). OR-2 and WA-2 had the lowest AIC values based on the SDM model; however, there were no significant effects of soil texture and elevation on *P. rubi* DNA in roots (*P* > 0.05) (Table 4).

**Table 4:** Coefficient estimates and SE for the OLS, SLM, SDM, SEM, CAR, and SARMA multiple regression models and associated VIF and *P* values for *Phytophthora rubi* DNA concentrations in roots regressed against soil texture (sand, silt, and clay) and elevation in four commercial red raspberry production fields in Oregon (OR-1 and OR-2) and Washington (WA-1 and WA-2).

**Field**	**Variables^a^**	**OLS**					**SLM**				**SDM**				**SEM**				**CAR**				**SARMA**			
					
		**Estimate**	**SE**	***P***	**VIF**	**Model**	**Estimate**	**SE**	***P***	**Model**	**Estimate**	**SE**	***P***	**Model**	**Estimate**	**SE**	***P***	**Model**	**Estimate**	**SE**	***P***	**Model**	**Estimate**	**SE**	***P***	**Model**
**OR-1**	Intercept	170.82	176.79	0.34	…	…	162.61	168.89	0.34	…	630.02	1,137.13	0.58	…	206.50	171.30	0.23	…	165.21	169.63	0.33	…	352.59	174.37	0.04	…
	Sand	−90.38	93.71	0.34	834	…	−86.08	89.52	0.34	…	−58.62	85.97	0.50	…	−109.83	90.71	0.23	…	−87.16	89.91	0.33	…	−190.22	91.85	0.04	…
	Silt	−106.57	106.04	0.32	672	…	−101.50	101.30	0.32	….	−69.18	97.38	0.48	….	−129.10	102.67	0.21	….	−103.13	101.73	0.31	….	−219.04	104.35	0.04	….
	Clay	−71.96	84.94	0.40	371	….	−68.82	81.13	0.40	….	−43.99	78.40	0.57	….	−86.67	82.50	0.29	….	−69.55	81.53	0.39	….	−150.11	84.75	0.08	….
	Elevation	0.14	0.21	0.52	1	….	0.14	0.21	0.51	….	−0.27	0.56	0.63	….	0.18	0.16	0.26	….	0.12	0.20	0.55	….	0.34	0.07	<0.001	….
	*P*	….	….	….	….	0.12	….	….	….	0.61	….	….	….	0.002	….	….	….	0.52	….	….	….	0.81	….	….	….	<0.001
	*R* ^2^	….	….	….	….	0.12	….	….	….	0.13	….	….	….	0.31	….	….	….	0.13	….	….	….	0.12	….	….	….	0.27
	ρ	….	….	….	….	….	….	….	….	0.16	….	….	….	−1.61	….	….	….	….	….	….	….	….	….	….	….	….
	λ	….	….	….	….	….	….	….	….	….	….	….	….	….	….	….	….	−0.45	….	….	….	−0.11	….	….	….	−1.00
	AIC	….	….	….	….	188.75	….	….	….	190.52	….	….	….	184.66	….	….	….	190.33	….	….	….	190.69	….	….	….	179.81

**OR-2**	Intercept	3.57	5.96	0.55	….	….	3.77	5.69	0.51	….	64.68	34.00	0.06	….	3.71	5.71	0.52	….	3.81	5.71	0.50	….	3.75	5.71	0.51	….
	Sand	−3.33	3.36	0.33	7	….	−3.35	3.21	0.30	….	−4.27	2.96	0.15	….	−3.29	3.21	0.30	….	−3.31	3.21	0.30	….	−3.25	3.20	0.31	….
	Silt	−1.35	2.73	0.62	4	….	−1.39	2.61	0.59	….	−0.90	2.37	0.71	….	−1.35	2.62	0.61	….	−1.33	2.62	0.61	….	−1.33	2.62	0.61	….
	Clay	−1.74	4.84	0.72	4	….	−1.95	4.63	0.67	….	−4.48	4.31	0.30	….	−2.11	4.64	0.65	….	−2.24	4.64	0.63	….	−2.28	4.65	0.62	….
	Elevation	0.70	0.43	0.11	1	….	0.72	0.41	0.08	….	0.99	0.43	0.02	….	0.74	0.41	0.07	….	0.71	0.41	0.08	….	0.77	0.41	0.06	….
	*P*	….	….	….	….	0.20	….	….	….	0.66	….	….	….	0.08	….	….	….	0.75	….	….	….	0.80	….	….	….	0.70
	*R* ^2^	….	….	….	….	0.10	….	….	….	0.10	….	….	….	0.26	….	….	….	0.10	….	….	….	0.10	….	….	….	0.10
	ρ	….	….	….	….	….	….	….	….	−0.19	….	….	….	−0.95	….	….	….	….	….	….	….	….	….	….	….	….
	λ	….	….	….	….	….	….	….	….	….	….	….	….	….	….	….	….	−0.16	….	….	….	−0.21	….	….	….	−0.22
	AIC	….	….	….	….	140.22	….	….	….	142.03	….	….	….	138.41	….	….	….	142.12	….	….	….	142.16	….	….	….	142.07

**WA-1**	Intercept	377.42	178.96	0.04	….	….	366.48	171.24	0.03	….	2,253.52	928.36	0.02	….	433.58	172.67	0.01	….	394.63	172.45	0.02	….	623.12	168.46	<0.001	….
	Sand	−234.25	110.15	0.04	2,273	….	−227.56	105.39	0.03	….	−307.03	109.63	0.01	….	−269.09	106.27	0.01	….	−245.12	106.14	0.02	….	−385.75	103.65	<0.001	….
	Silt	−216.77	102.23	0.04	1,513	….	−210.62	97.81	0.03	….	−283.21	101.50	0.01	….	−249.03	98.70	0.01	….	−226.96	98.56	0.02	….	−359.67	96.37	<0.001	….
	Clay	−136.93	69.69	0.05	234	….	−132.92	66.68	0.05	….	−182.98	69.40	0.01	….	−157.58	67.22	0.02	….	−142.08	67.14	0.03	….	−226.26	65.59	<0.001	….
	Elevation	0.16	0.59	0.79	2	….	0.23	0.59	0.70	….	0.61	0.96	0.53	….	0.16	0.53	0.76	….	0.14	0.54	0.80	….	0.03	0.47	0.95	….
	*P*	….	….	….	….	0.06	….	….	….	0.79	….	….	….	0.32	….	….	….	0.56	….	….	….	0.64	….	….	….	<0.001
	*R* ^2^	….	….	….	….	0.15	….	….	….	0.15	….	….	….	0.26	….	….	….	0.16	….	….	….	0.16	….	….	….	0.33
	ρ	….	….	….	….	….	….	….	….	0.10	….	….	….	−0.56	….	….	….	….	….	….	….	….	….	….	….	….
	λ	….	….	….	….	….	….	….	….	….	….	….	….	….	….	….	….	−0.35	….	….	….	−0.47	….	….	….	−1.00
	AIC	….	….	….	….	205.23	….	….	….	207.16	….	….	….	207.30	….	….	….	206.89	….	….	….	207.01	….	….	….	192.91

**WA-2**	Intercept	31.13	81.32	0.70	….	….	55.75	74.27	0.45	….	−1,283.90	435.02	0.003	….	81.42	72.40	0.26	….	73.91	75.04	0.32	….	81.12	68.18	0.23	….
	Sand	−13.68	46.87	0.77	918	….	−29.74	42.81	0.49	….	1.43	45.08	0.97	….	−44.84	41.77	0.28	….	−39.77	43.26	0.36	….	−45.34	39.32	0.25	….
	Silt	−20.09	48.30	0.68	740	….	−35.34	44.11	0.42	….	−3.13	46.18	0.95	….	−50.61	43.02	0.24	….	−46.71	44.59	0.29	….	−50.56	40.54	0.21	….
	Clay	−14.28	35.07	0.69	150	….	−22.02	32.03	0.49	….	7.21	33.75	0.83	….	−31.23	31.03	0.31	….	−29.04	32.27	0.37	….	−29.98	29.13	0.30	….
	Elevation	0.22	0.41	0.60	1	….	0.07	0.38	0.86	….	0.36	0.66	0.58	….	0.21	0.55	0.71	….	0.47	0.49	0.34	….	0.30	0.59	0.61	….
	*P*	….	….	….	….	0.01	….	….	….	0.04	….	….	….	0.72	….	….	….	0.05	….	….	….	0.16	….	….	….	0.01
	*R* ^2^	….	….	….	….	0.21	….	….	….	0.26	….	….	….	0.39	….	….	….	0.26	….	….	….	0.24	….	….	….	0.29
	ρ	….	….	….	….	….	….	….	….	0.56	….	….	….	−0.17	….	….	….	….	….	….	….	….	….	….	….	….
	λ	….	….	….	….	….	….	….	….	….	….	….	….	….	….	….	….	0.70	….	….	….	0.76	….	….	….	1.82
	AIC	….	….	….	….	174.41	….	….	….	172.22	….	….	….	168.70	….	….	….	172.43	….	….	….	174.42	….	….	….	170.42

a Independent variables were sand, silt, clay, and field elevation. The *P* value represents the significance of the model at *P* < 0.05. *R*^2^ is the Nagelkerke pseudo-R-squared; ρ is a fitting parameter for SLM and SDM; λ is a fitting parameter for SEM, CAR, and SARMA. AIC was used to compare OLS, SLM, SDM, SEM, CAR, and SARMA models and determine the best model that fits the data for each field. The lower the AIC, the better the model.

AIC, Akaike information criterion; CAR, conditional autoregressive; OLS, ordinary least squares; P, probability; SARMA, spatial autoregressive moving average; SDM, spatial Durbin model; SE, standard errors; SEM, spatial error model; SLM, spatial lag model; VIF, variance inflation factor.

*Pratylenchus penetrans* densities in roots were not significantly influenced by soil texture and field elevation in any of the fields when using OLS multivariate regression analysis (*P* > 0.05) (Table 5). Severe multicollinearity effects were observed for soil texture across all fields except OR-2. No significant differences were observed for the spatial autocorrelation in the residuals for all fields (*P* > 0.05) (Table S1 in Supplementary Materials). For OR-1 and WA-2, the SARMA model revealed significant effects of field elevation on the distribution of *P. penetrans* densities in roots (*P* < 0.05; AIC = 0.31, *R*^2^ = 0.42). For OR-2 and WA-1, the OLS model had the lowest AIC values compared to the other models; however, soil texture and elevation had no significant influence on *P. penetrans* densities in roots (*P* > 0.05) (Table 5). *P. penetrans* densities in soil was not significantly influenced by soil texture and field elevation in none of the fields when using OLS (*P* > 0.05), and severe multicollinearity effects were observed across all fields except OR-2 (Table 6). Spatial autocorrelation in the residuals was significant for OR-2, WA-1, and WA-2 (*P* < 0.05) (Table S1 in Supplementary Materials). WA-1 was the only field in which sand, silt, and clay had a significant effect on *P. penetrans* densities in soil using the SDM model (*P* < 0.05; AIC = 111.71, *R*^2^ = 0.59) (Table 6).

**Table 5:** Coefficient estimates and SE for the OLS, SLM, SDM, SEM, CAR, and SARMA multiple regression models and associated VIF and *P* values for *Pratylenchus penetrans* densites in roots regressed against soil texture (sand, silt, and clay) and elevation in four commercial red raspberry production fields in Oregon (OR-1 and OR-2) and Washington (WA-1 and WA-2).

**Field**	**Variables^a^**	**OLS**					**SLM**				**SDM**				**SEM**				**CAR**				**SARMA**			
					
		**Estimate**	**SE**	***P***	**VIF**	**Model**	**Estimate**	**SE**	***P***	**Model**	**Estimate**	**SE**	***P***	**Model**	**Estimate**	**SE**	***P***	**Model**	**Estimate**	**SE**	***P***	**Model**	**Estimate**	**SE**	***P***	**Model**
**OR-1**	Intercept	22.66	119.13	0.85	….	….	22.73	114.06	0.84	….	973.21	805.65	0.23	….	29.70	115.29	0.80	….	28.07	114.95	0.80	….	−0.03	111.84	0.99	….
	Sand	−12.03	63.15	0.85	834	….	−12.05	60.46	0.84	….	−57.46	62.92	0.36	….	−15.45	61.03	0.80	….	−14.49	60.60	0.81	….	1.24	58.91	0.98	….
	Silt	−10.88	71.46	0.88	672	….	−10.86	68.42	0.87	….	−62.92	71.27	0.38	….	−14.67	69.08	0.83	….	−13.61	68.92	0.84	….	3.39	66.93	0.96	….
	Clay	−14.89	57.24	0.80	371	….	−15.03	54.80	0.78	….	−55.89	57.40	0.33	….	−19.50	55.57	0.73	….	−18.83	55.31	0.73	….	−6.89	54.35	0.90	….
	Elevation	0.10	0.14	0.50	1	….	0.11	0.14	0.44	….	−0.34	0.41	0.42	….	0.12	0.10	0.22	….	0.09	0.12	0.46	….	0.15	0.05	0.002	….
	*P*	….	….	….	….	0.37	….	….	….	0.87	….	….	….	0.14	….	….	….	0.35	….	….	….	0.52	….	….	….	<0.001
	*R* ^2^	….	….	….	….	0.07	….	….	….	0.07	….	….	….	0.18	….	….	….	0.09	….	….	….	0.08	….	….	….	0.31
	ρ	….	….	….	….	….	….	….	….	−0.07	….	….	….	−0.88	….	….	….	….	….	….	….	….	….	….	….	….
	λ	….	….	….	….	….	….	….	….	….	….	….	….	….	….	….	….	−0.60	….	….	….	−0.58	….	….	….	−0.99
	AIC	….	….	….	….	141.38	….	….	….	143.35	….	….	….	143.80	….	….	….	142.50	….	….	….	142.96	….	….	….	125.53

**OR-2**	Intercept	3.49	10.82	0.75	….	….	3.60	10.36	0.73	….	25.86	63.55	0.68	….	3.27	10.36	0.75	….	3.77	10.36	0.72	….	3.34	10.35	0.75	….
	Sand	0.35	6.10	0.95	7	….	0.44	5.84	0.94	….	−1.02	5.62	0.86	….	0.83	5.81	0.89	….	0.60	5.83	0.92	….	1.23	5.76	0.83	….
	Silt	−3.42	4.96	0.49	4	….	−3.41	4.74	0.47	….	−3.60	4.49	0.42	….	−3.35	4.76	0.48	….	−3.38	4.75	0.48	….	−3.45	4.77	0.47	….
	Clay	1.80	8.80	0.84	4	….	1.71	8.42	0.84	….	−0.04	8.11	0.99	….	1.55	8.43	0.85	….	0.99	8.43	0.91	….	1.10	8.46	0.90	….
	Elevation	−0.92	0.79	0.25	1	….	−0.90	0.75	0.23	….	−1.18	0.80	0.14	….	−0.88	0.74	0.24	….	−0.94	0.75	0.21	….	−0.88	0.72	0.22	….
	*P*	….	….	….	….	0.49	….	….	….	0.80	….	….	….	0.19	….	….	….	0.66	….	….	….	0.77	….	….	….	0.51
	*R* ^2^	….	….	….	….	0.06	….	….	….	0.06	….	….	….	0.17	….	….	….	0.06	….	….	….	0.06	….	….	….	0.07
	ρ	….	….	….	….	….	….	….	….	−0.10	….	….	….	−0.64	….	….	….	….	….	….	….	….	….	….	….	….
	λ	….	….	….	….	….	….	….	….	….	….	….	….	….	….	….	….	−0.22	….	….	….	−0.18	….	….	….	−0.43
	AIC	….	….	….	….	211.88	….	….	….	213.82	….	….	….	214.17	….	….	….	213.68	….	….	….	213.80	….	….	….	213.44

**WA-1**	Intercept	−76.26	100.24	0.45	….	….	−77.12	95.99	0.42	….	179.96	501.46	0.72	….	−76.26	96.02	0.43	….	−76.81	96.01	0.42	….	−76.27	96.05	0.43	….
	Sand	46.61	61.70	0.45	2,272	….	47.17	59.08	0.42	….	36.20	62.66	0.56	….	46.62	59.10	0.43	….	46.94	59.09	0.43	….	46.62	59.12	0.43	….
	Silt	44.13	57.26	0.44	1,513	….	44.59	54.83	0.42	….	33.18	58.03	0.57	….	44.12	54.85	0.42	….	44.43	54.84	0.42	….	44.11	54.87	0.42	….
	Clay	29.62	39.04	0.45	234	….	30.00	37.38	0.42	….	22.51	39.73	0.57	….	29.63	37.39	0.43	….	29.86	37.39	0.42	….	29.64	37.41	0.43	….
	Elevation	0.39	0.33	0.24	2	….	0.40	0.32	0.22	….	0.23	0.56	0.68	….	0.39	0.32	0.21	….	0.39	0.32	0.21	….	0.39	0.32	0.21	….
	*P*	….	….	….	….	0.58	….	….	….	0.88	….	….	….	0.57	….	….	….	0.98	….	….	….	0.99	….	….	….	0.97
	*R* ^2^	….	….	….	….	0.05	….	….	….	0.05	….	….	….	0.12	….	….	….	0.05	….	….	….	0.05	….	….	….	0.05
	ρ	….	….	….	….	….	….	….	….	−0.07	….	….	….	−0.25	….	….	….	….	….	….	….	….	….	….	….	….
	λ	….	….	….	….	….	….	….	….	….	….	….	….	….	….	….	….	−0.01	….	….	….	−0.02	….	….	….	−0.02
	AIC	….	….	….	….	135.68	….	….	….	137.66	….	….	….	141.14	….	….	….	137.68	….	….	….	137.68	….	….	….	137.68

**WA-2**	Intercept	−69.62	92.71	0.46	….	….	−80.33	88.63	0.36	….	−555.77	494.67	0.26	….	−85.09	83.81	0.31	….	−105.32	88.00	0.23	….	−102.97	81.30	0.20	….
	Sand	42.80	53.43	0.43	918	….	49.75	51.18	0.33	….	49.42	51.62	0.34	….	52.09	48.36	0.28	….	63.01	50.72	0.21	….	62.42	46.93	0.18	….
	Silt	43.95	55.07	0.43	740	….	50.55	52.68	0.34	….	52.80	52.88	0.32	….	52.72	49.63	0.29	….	65.84	52.22	0.21	….	63.45	48.10	0.19	….
	Clay	23.00	39.98	0.57	150	….	26.83	38.16	0.48	….	31.98	38.73	0.41	….	29.20	36.41	0.42	….	37.54	38.05	0.32	….	36.09	35.40	0.31	….
	Elevation	0.81	0.47	0.09	1	….	1	0.47	0.03	….	0.24	0.77	0.75	….	1.04	0.26	<0.001	….	0.81	0.37	0.03	….	1.19	0.17	<0.001	….
	*P*	….	….	….	….	0.03	….	….	….	0.45	….	….	….	0.02	….	….	….	0.04	….	….	….	0.23	….	….	….	<0.001
	*R* ^2^	….	….	….	….	0.18	….	….	….	0.19	….	….	….	0.29	….	….	….	0.24	….	….	….	0.20	….	….	….	0.42
	ρ	….	….	….	….	….	….	….	….	−0.29	….	….	….	−1.29	….	….	….	….	….	….	….	….	….	….	….	….
	λ	….	….	….	….	….	….	….	….	….	….	….	….	….	….	….	….	−1.25	….	….	….	−0.87	….	….	….	−0.99
	AIC	….	….	….	….	190.14	….	….	….	191.57	….	….	….	191.06	….	….	….	187.75	….	….	….	190.73	….	….	….	170.95

a Independent variables were sand, silt, clay, and field elevation. The *P* value represents the significance of the model at *P* < 0.05. *R*^2^ is the Nagelkerke pseudo-R-squared; ρ is a fitting parameter for SLM and SDM; λ is a fitting parameter for SEM, CAR, and SARMA. AIC was used to compare OLS, SLM, SDM, SEM, CAR, and SARMA models and determine the best model that fits the data for each field. The lower the AIC, the better the model.

AIC, Akaike information criterion; CAR, conditional autoregressive; OLS, ordinary least squares; *P*, probability; SARMA, spatial autoregressive moving average; SDM, spatial Durbin model; SE, standard errors; SEM, spatial error model; SLM, spatial lag model; VIF, variance inflation factor.

**Table 6:** Coefficient estimates and SE for the OLS, SLM, SDM, SEM, CAR, and SARMA multiple regression models and associated VIF and *P* values for *Pratylenchus penetrans* in soil regressed against soil texture (sand, silt, and clay) and elevation in four commercial red raspberry production fields in Oregon (OR-1 and OR-2) and Washington (WA-1 and WA-2).

**Field**	**Variables^a^**	**OLS**					**SLM**				**SDM**				**SEM**				**CAR**				**SARMA**			
					
		**Estimate**	**SE**	***P***	**VIF**	**Model**	**Estimate**	**SE**	***P***	**Model**	**Estimate**	**SE**	***P***	**Model**	**Estimate**	**SE**	***P***	**Model**	**Estimate**	**SE**	***P***	**Model**	**Estimate**	**SE**	***P***	**Model**
**OR-1**	Intercept	−36.41	148.53	0.80	….	….	−37.35	142.21	0.79	….	1,044.05	848.43	0.22	….	−68.40	143.65	0.63	….	−52.73	143.26	0.71	….	−71.80	144.06	0.62	….
	Sand	19.96	78.73	0.80	834	….	20.50	75.38	0.78	….	−70.87	63.18	0.26	….	38.28	76.03	0.61	….	29.76	75.90	0.69	….	39.88	76.26	0.60	….
	Silt	25.04	89.09	0.78	672	….	25.66	85.30	0.76	….	−78.09	71.53	0.27	….	45.24	86.07	0.60	….	35.89	85.89	0.68	….	47.07	86.33	0.59	….
	Clay	12.55	71.36	0.86	371	….	12.91	68.32	0.85	….	−70.68	57.59	0.22	….	24.91	69.27	0.72	….	17.65	68.94	0.80	….	27.04	69.43	0.70	….
	Elevation	0.13	0.18	0.48	1	….	0.13	0.18	0.46	….	0.82	0.42	0.05	….	0.06	0.12	0.61	….	0.04	0.15	0.77	….	0.08	0.12	0.53	….
	*P*	….	….	….	….	0.36	….	….	….	0.92	….	….	….	<0.001	….	….	….	0.31	….	….	….	0.48	….	….	….	0.37
	*R* ^2^	….	….	….	….	0.07	….	….	….	0.07	….	….	….	0.42	….	….	….	0.09	….	….	….	0.08	….	….	….	0.09
	ρ	….	….	….	….	….	….	….	….	−0.03	….	….	….	−1.95	….	….	….	….	….	….	….	….	….	….	….	….
	λ	….	….	….	….	….	….	….	….	….	….	….	….	….	….	….	….	−0.67	….	….	….	−0.60	….	….	….	−0.43
	AIC	….	….	….	….	167.85	….	….	….	169.84	….	….	….	149.76	….	….	….	168.8	….	….	….	169.35	….	….	….	169.04

**OR-2**	Intercept	0.34	5.17	0.95	….	….	−0.45	4.92	0.93	….	15.33	31.00	0.62	….	−0.02	4.87	0.99	….	0.03	4.94	0.99	….	−0.01	4.88	0.99	….
	Sand	0.76	2.92	0.80	7	….	0.87	2.76	0.75	….	−0.32	2.74	0.91	….	0.82	2.77	0.77	….	0.69	2.79	0.80	….	0.79	2.77	0.78	….
	Silt	−0.36	2.37	0.88	4	….	−0.15	2.24	0.94	….	−1.28	2.21	0.56	….	−0.02	2.22	0.99	….	−0.34	2.26	0.88	….	−0.01	2.23	0.99	….
	Clay	3.04	4.20	0.47	4	….	3.13	3.98	0.43	….	1.99	3.95	0.61	….	3.10	3.96	0.43	….	3.60	4.01	0.37	….	3.13	3.97	0.43	….
	Elevation	−0.53	0.38	0.16	1	….	−0.49	0.36	0.16	….	−0.84	0.40	0.03	….	−0.49	0.36	0.18	….	−0.49	0.36	0.18	….	−0.50	0.36	0.17	….
	*P*	….	….	….	….	0.64	….	….	….	0.32	….	….	….	0.95	….	….	….	0.35	….	….	….	0.69	….	….	….	0.37
	*R* ^2^	….	….	….	….	0.04	….	….	….	0.06	….	….	….	0.14	….	….	….	0.06	….	….	….	0.05	….	….	….	0.06
	ρ	….	….	….	….	….	….	….	….	0.29	….	….	….	−0.03	….	….	….	….	….	….	….	….	….	….	….	….
	λ	….	….	….	….	….	….	….	….	….	….	….	….	….	….	….	….	0.30	….	….	….	0.11	….	….	….	0.34
	AIC	….	….	….	….	123.25	….	….	….	124.26	….	….	….	126.93	….	….	….	124.40	….	….	….	125.09	….	….	….	124.45

**WA-1**	Intercept	30.66	99.00	0.76	….	….	13.45	86.71	0.88	….	2,135.04	424.48	<0.001	….	−33.96	86.30	0.69	….	−4.70	90.26	0.96	….	−58.12	84.84	0.49	….
	Sand	−17.59	60.93	0.77	2,273	….	−7.27	53.37	0.89	….	−140.67	52.15	0.007	….	21.97	53.10	0.68	….	4.29	55.54	0.93	….	37.00	52.21	0.48	….
	Silt	−17.61	56.55	0.76	1,513	….	−7.56	49.53	0.88	….	−132.37	48.37	0.006	….	19.72	49.16	0.69	….	2.66	51.48	0.96	….	33.30	48.34	0.49	….
	Clay	−15.32	38.55	0.69	234	….	−8.53	33.77	0.80	….	−93.85	33.09	0.004	….	10.04	33.65	0.77	….	−2.33	35.17	0.95	….	19.54	33.11	0.55	….
	Elevation	0.68	0.33	0.04	2	….	0.22	0.30	0.47	….	−0.63	0.44	0.15	….	0.38	0.39	0.33	….	0.49	0.35	0.16	….	0.45	0.37	0.22	….
	*P*	….	….	….	….	<0.001	….	….	….	0.004	….	….	….	0.96	….	….	….	0.02	….	….	….	0.07	….	….	….	0.02
	*R* ^2^	….	….	….	….	0.29	….	….	….	0.38	….	….	….	0.59	….	….	….	0.35	….	….	….	0.32	….	….	….	0.35
	ρ	….	….	….	….	….	….	….	….	0.70	….	….	….	−0.02	….	….	….	….	….	….	….	….	….	….	….	….
	λ	….	….	….	….	….	….	….	….	….	….	….	….	….	….	….	….	0.72	….	….	….	0.77	….	….	….	1.01
	AIC	….	….	….	….	134.18	….	….	….	127.78	….	….	….	111.71	….	….	….	130.76	….	….	….	133.03	….	….	….	130.58

**WA-2**	Intercept	−60.06	53.39	0.26	….	….	−51.53	49.99	0.30	….	157.38	292.91	0.59	….	−52.72	49.96	0.29	….	−55.47	50.96	0.28	….	−40.27	49.21	0.41	….
	Sand	36.21	30.77	0.24	918	….	30.52	28.82	0.29	….	5.95	30.84	0.85	….	31.58	28.80	0.27	….	33.65	29.37	0.25	….	24.24	28.37	0.39	….
	Silt	38.69	31.71	0.23	740	….	32.91	29.70	0.27	….	9.24	31.60	0.77	….	34.22	29.69	0.25	….	35.80	30.27	0.24	….	26.65	29.25	0.36	….
	Clay	22.07	23.02	0.34	150	….	18.93	21.55	0.38	….	3.47	23.16	0.88	….	20.09	21.48	0.35	….	20.22	21.96	0.36	….	15.37	21.12	0.47	….
	Elevation	−0.03	0.27	0.92	1	….	−0.04	0.25	0.87	….	−0.39	0.46	0.39	….	−0.11	0.32	0.73	….	0.003	0.27	0.99	….	−0.12	0.34	0.73	….
	*P*	….	….	….	….	0.08	….	….	….	0.17	….	….	….	0.74	….	….	….	0.38	….	….	….	0.71	….	….	….	0.24
	*R* ^2^	….	….	….	….	0.15	….	….	….	0.17	….	….	….	0.25	….	….	….	0.16	….	….	….	0.15	….	….	….	0.16
	ρ	….	….	….	….	….	….	….	….	0.45	….	….	….	0.14	….	….	….	….	….	….	….	….	….	….	….	….
	λ	….	….	….	….	….	….	….	….	….	….	….	….	….	….	….	….	0.42	….	….	….	0.15	….	….	….	0.83
	AIC	….	….	….	….	123.91	….	….	….	124.05	….	….	….	126.10	….	….	….	125.13	….	….	….	125.77	….	….	….	124.55

a Independent variables were sand, silt, clay, and field elevation. The *P* value represents the significance of the model at *P* < 0.05. *R*^2^ is the Nagelkerke pseudo-R-squared; ρ is a fitting parameter for SLM and SDM; λ is a fitting parameter for SEM, CAR, and SARMA. AIC was used to compare OLS, SLM, SDM, SEM, CAR, and SARMA models and determine the best model that fits the data for each field. The lower the AIC, the better the model.

AIC, Akaike information criterion; CAR, conditional autoregressive; OLS, ordinary least squares; *P*, probability; SARMA, spatial autoregressive moving average; SDM, spatial Durbin model; SE, standard errors; SEM, spatial error model; SLM, spatial lag model; VIF, variance inflation factor.

### Effects of *P. rubi* and *P. penetrans* on disease severity

Disease severity was significantly influenced by *P. rubi* and *P. penetrans* in soil for OR-1, WA-1, and WA-2 (*P* < 0.05) in the OLS model, and no severe multicollinearity effects were observed (Table 7). However, spatial autocorrelation in the residuals was significant for OR-1, WA-1, and WA-2 (*P* < 0.05) (Table S2 in Supplementary Materials). For OR-1 and WA-2, the SDM model had the lowest AIC values, −96.37 and −98.44, respectively, and *P. rubi* significantly affected disease severity (*P* < 0.05) with *R*^2^ values, 0.61 and 0.73, respectively. Additionally, *P. penetrans* in soil had a significant effect on disease severity in WA-2 (*P* < 0.05). For OR-2, the OLS model had the lowest AIC values, −153.46, compared to the other models. However, no significant effects of *P. rubi* and *P. penetrans* were observed (*P* > 0.05). Finally, for WA-1, the SLM model had the lowest AIC values, −56.27, and *P. rubi* had a significant effect on disease severity (*P* < 0.05) with an *R*^2^ value of 0.43 (Table 7).

**Table 7:** Coefficient estimates and SE for the OLS, SLM, SDM, SEM, CAR, and SARMA multiple regression models and associated VIF and *P* values for disease rating regressed against *Phytophthora rubi* DNA in roots, *Pratylenchus penetrans* in roots and soil in four commercial red raspberry production fields in Oregon (OR-1 and OR-2) and Washington (WA-1 and WA-2).

**Field**	**Variables^a^**	**OLS**					**SLM**				**SDM**				**SEM**				**CAR**				**SARMA**			
					
		**Estimate**	**SE**	***P***	**VIF**	**Model**	**Estimate**	**SE**	***P***	**Model**	**Estimate**	**SE**	***P***	**Model**	**Estimate**	**SE**	***P***	**Model**	**Estimate**	**SE**	***P***	**Model**	**Estimate**	**SE**	***P***	**Model**
**OR-1**	Intercept	0.83	0.03	<0.001	….	….	0.20	0.14	0.15	….	0.78	0.34	0.02	….	0.83	0.05	<0.001	….	0.82	0.03	<0.001	….	0.83	0.05	<0.001	….
	*P. rubi* DNA	0.08	0.01	<0.001	1.02	….	0.07	0.01	<0.001	….	0.07	0.01	<0.001	….	0.07	0.01	<0.001	….	0.09	0.01	<0.001	….	0.07	0.01	<0.001	….
	*P. penetrans* (root)	0.01	0.02	0.57	1.30	….	0.01	0.02	0.52	….	0.02	0.02	0.20	….	0.01	0.02	0.62	….	0.01	0.02	0.71	….	0.01	0.02	0.66	….
	*P. penetrans* (soil)	−0.04	0.02	0.04	1.32	….	−0.02	0.02	0.15	….	−0.03	0.02	0.10	….	−0.02	0.02	0.19	….	−0.03	0.02	0.04	….	−0.01	0.01	0.42	….
	*P*	….	….	….	….	<0.001	….	….	….	<0.001	….	….	….	0.79	….	….	….	<0.001	….	….	….	0.56	….	….	….	<0.001
	*R* ^2^	….	….	….	….	0.46	….	….	….	0.57	….	….	….	0.61	….	….	….	0.53	….	….	….	0.46	….	….	….	0.56
	ρ	….	….	….	….	….	….	….	….	0.67	….	….	….	−0.12	….	….	….	….	….	….	….	….	….	….	….	….
	λ	….	….	….	….	….	….	….	….	….	….	….	….	….	….	….	….	0.73	….	….	….	0.09	….	….	….	2.06
	AIC	….	….	….	….	−84.58	….	….	….	−95.82	….	….	….	−96.37	….	….	….	−90.26	….	….	….	−82.92	….	….	….	−95.25

**OR-2**	Intercept	0.61	0.02	<0.001	….	….	0.58	0.23	0.01	….	0.74	0.25	0.003	….	0.61	0.02	<0.001	….	0.61	0.02	<0.001	….	0.61	0.02	<0.001	….
	*P. rubi* DNA	0.01	0.01	0.27	1.01	….	0.01	0.01	0.25	….	0.006	0.01	0.56	….	0.01	0.01	0.25	….	0.01	0.01	0.26	….	0.01	0.01	0.25	….
	*P. penetrans* (root)	0.008	0.006	0.22	1.09	….	0.008	0.006	0.21	….	0.01	0.006	0.08	….	0.008	0.006	0.18	….	0.008	0.006	0.19	….	0.008	0.006	0.18	….
	*P. penetrans* (soil)	0.01	0.01	0.38	1.09	….	0.01	0.01	0.36	….	0.004	0.01	0.74	….	0.01	0.01	0.35	….	0.01	0.01	0.35	….	0.01	0.01	0.34	….
	*P*	….	….	….	….	0.21	….	….	….	0.88	….	….	….	0.58	….	….	….	0.81	….	….	….	0.95	….	….	….	0.77
	*R* ^2^	….	….	….	….	0.08	….	….	….	0.08	….	….	….	0.17	….	….	….	0.08	….	….	….	0.08	….	….	….	0.08
	ρ	….	….	….	….	….	….	….	….	0.05	….	….	….	−0.24	….	….	….	….	….	….	….	….	….	….	….	….
	λ	….	….	….	….	….	….	….	….	….	….	….	….	….	….	….	….	−0.10	….	….	….	−0.01	….	….	….	−0.13
	AIC	….	….	….	….	−153.46	….	….	….	−151.48	….	….	….	−152.09	….	….	….	−151.52	….	….	….	−151.47	….	….	….	−151.54

**WA-1**	Intercept	0.59	0.03	<0.001	….	….	0.31	0.13	0.01	….	0.82	0.27	0.003	….	0.58	0.04	<0.001	….	0.59	0.03	<0.001	….	0.58	0.04	<0.001	….
	*P. rubi* DNA	0.09	0.01	<0.001	1.00	….	0.08	0.01	<0.001	….	0.08	0.01	<0.001	….	0.08	0.01	<0.001	….	0.09	0.01	<0.001	….	0.08	0.01	<0.001	….
	*P. penetrans* (root)	−0.01	0.03	0.63	1.01	….	−0.008	0.02	0.74	….	−0.02	0.02	0.48	….	−0.009	0.02	0.74	….	−0.01	0.03	0.59	….	−0.008	0.02	0.73	….
	*P. penetrans* (soil)	−0.05	0.02	0.03	1.01	….	−0.03	0.02	0.18	….	−0.02	0.03	0.51	….	−0.04	0.02	0.11	….	−0.05	0.02	0.04	….	−0.038	0.03	0.14	….
	*P*	….	….	….	….	<0.001	….	….	….	0.04	….	….	….	0.36	….	….	….	0.35	….	….	….	0.83	….	….	….	0.28
	*R* ^2^	….	….	….	….	0.43	….	….	….	0.46	….	….	….	0.51	….	….	….	0.43	….	….	….	0.43	….	….	….	0.44
	ρ	….	….	….	….	….	….	….	….	0.44	….	….	….	−0.48	….	….	….	….	….	….	….	….	….	….	….	….
	λ	….	….	….	….	….	….	….	….	….	….	….	….	….	….	….	….	0.38	….	….	….	0.04	….	….	….	0.63
	AIC	….	….	….	….	−54.35	….	….	….	−56.27	….	….	….	−55.19	….	….	….	−53.22	….	….	….	−52.40	….	….	….	−53.52

**WA-2**	Intercept	0.53	0.05	<0.001	….	….	0.09	0.09	0.27	….	0.47	0.18	0.009	….	0.59	0.09	<0.001	….	0.57	0.05	<0.001	….	0.62	0.06	<0.001	….
	*P. rubi* DNA	0.12	0.01	<0.001	1.07	….	0.08	0.01	<0.001	….	0.08	0.01	<0.001	….	0.08	0.01	<0.001	….	0.12	0.01	<0.001	….	0.08	0.01	<0.001	….
	*P. penetrans* (root)	0.01	0.01	0.44	1.14	….	−0.009	0.01	0.39	….	−0.01	0.01	0.22	….	−0.009	0.01	0.40	….	0.01	0.01	0.44	….	−0.01	0.01	0.20	….
	*P. penetrans* (soil)	0.07	0.02	0.006	1.08	….	0.04	0.02	0.02	….	0.05	0.02	0.009	….	0.05	0.02	0.01	….	0.04	0.02	0.08	….	0.07	0.02	<0.001	….
	*P*	….	….	….	….	<0.001	….	….	….	<0.001	….	….	….	0.60	….	….	….	<0.001	….	….	….	0.18	….	….	….	<0.001
	*R* ^2^	….	….	….	….	0.57	….	….	….	0.70	….	….	….	0.73	….	….	….	0.68	….	….	….	0.58	….	….	….	0.68
	ρ	….	….	….	….	….	….	….	….	0.67	….	….	….	0.20	….	….	….	….	….	….	….	….	….	….	….	….
	λ	….	….	….	….	….	….	….	….	….	….	….	….	….	….	….	….	0.84	….	….	….	0.31	….	….	….	2.06
	AIC	….	….	….	….	−76.74	….	….	….	−97.91	….	….	….	−98.44	….	….	….	−92.92	….	….	….	−76.50	….	….	….	−93.94

a Independent variables were *P. rubi* DNA concentrations in roots and *P. penetrans* in roots and soil. The *P* value represents the significance of the model at *P* < 0.05. *R*^2^ is the Nagelkerke pseudo-R-squared; *ρ* is a fitting parameter for SLM and SDM; λ is a fitting parameter for SEM, CAR, and SARMA. AIC was used to compare OLS, SLM, SDM, SEM, CAR, and SARMA models and determine the best model that fits the data for each field. The lower the AIC, the better the model.

AIC, Akaike information criterion; CAR, conditional autoregressive; OLS, ordinary least squares; *P*, probability; SARMA, spatial autoregressive moving average; SDM, spatial Durbin model; SE, standard errors; SEM, spatial error model; SLM, spatial lag model; VIF, variance inflation factor.

### Effects of *P. penetrans* in soil and *P. rubi* on *P. penetrans* infestation in roots

*Pratylenchus penetrans* densities in roots were significantly influenced by *P. penetrans* densitiesin soil for OR-1, OR-2, and WA-2 (*P* < 0.05) when using the OLS model, and no severe multicollinearity effects were observed (Table 8). No significant differences were observed for the spatial autocorrelation in the residuals across all fields (*P* > 0.05) (Table S3 in Supplementary Materials). For OR-1, the SLM and SARMA models had the same lowest AIC values, 128.26, and a similar *R*^2^ value of 0.23, with *P. penetrans* densities in soil significantly affecting *P. penetrans* in the roots (*P* < 0.05). For OR-2, the SARMA model had the lowest AIC value, 189.57, an *R*^2^ value of 0.33, and *P. penetrans* densities in the soil significantly affected *P. penetrans* in the roots (*P* < 0.05). For WA-1, the OLS model had the lowest AIC value, 134.05. However, *P. penetrans* densities in soil and *P. rubi* DNA concentrations in roots had no significant effect on *P. penetrans* densitiesin the roots (*P* > 0.05). For WA-2, the SDM model had the lowest AIC value, 183.53. However, *P. penetrans* densities in soil and *P. rubi* DNA concentrations in roots had no significant effect on *P. penetrans* densities in the roots (*P* > 0.05) (Table 8).

**Table 8:** Coefficient estimates and SE for the OLS, SLM, SDM, SEM, CAR, and SARMA multiple regression models and associated VIF and *P* values for *Pratylenchus penetrans* densities in roots regressed against *P. penetrans* densities in soil and *Phytophthora rubi* DNA concentrations in roots in four commercial red raspberry production fields in Oregon (OR-1 and OR-2) and Washington (WA-1 and WA-2).

**Field**	**Variables^a^**	**OLS**					**SLM**				**SDM**				**SEM**				**CAR**				**SARMA**			
					
		**Estimate**	**SE**	***P***	**VIF**	**Model**	**Estimate**	**SE**	***P***	**Model**	**Estimate**	**SE**	***P***	**Model**	**Estimate**	**SE**	***P***	**Model**	**Estimate**	**SE**	***P***	**Model**	**Estimate**	**SE**	***P***	**Model**
**OR-1**	Intercept	0.11	0.19	0.59	….	….	0.05	0.26	0.84	….	−1.19	1.28	0.35	….	0.10	0.19	0.60	….	0.11	0.19	0.57	….	0.09	0.19	0.62	….
	*P. penetrans* (soil)	0.39	0.09	<0.001	1.02	….	0.38	0.09	<0.001	….	0.42	0.10	<0.001	….	0.39	0.09	<0.001	….	0.39	0.09	<0.001	….	0.40	0.09	<0.001	….
	*P. rubi* DNA	0.02	0.08	0.82	1.02	….	0.02	0.07	0.79	….	0.06	0.08	0.46	….	0.02	0.07	0.83	….	0.01	0.07	0.87	….	0.02	0.07	0.83	….
	*P*	….	….	….	….	<0.001	….	….	….	0.75	….	….	….	0.56	….	….	….	0.83	….	….	….	0.85	….	….	….	0.75
	*R* ^2^	….	….	….	….	0.23	….	….	….	0.23	….	….	….	0.25	….	….	….	0.23	….	….	….	0.23	….	….	….	0.23
	ρ	….	….	….	….	….	….	….	….	0.11	….	….	….	−0.27	….	….	….	….	….	….	….	….	….	….	….	….
	λ	….	….	….	….	….	….	….	….	….	….	….	….	….	….	….	….	−0.10	….	….	….	−0.14	….	….	….	−0.18
	AIC	….	….	….	….	126.36	….	….	….	128.26	….	….	….	130.39	….	….	….	128.32	….	….	….	128.33	….	….	….	128.26

**OR-2**	Intercept	0.32	0.48	0.52	….	….	0.55	0.70	0.43	….	−3.38	1.69	0.04	….	0.19	0.44	0.66	….	0.07	0.44	0.86	….	−0.09	0.25	0.72	….
	*P. penetrans* (soil)	0.59	0.7	0.03	1.0	….	0.60	0.26	0.02	….	0.64	0.25	0.01	….	0.64	0.25	0.01	….	0.69	0.25	0.005	….	0.71	0.11	<0.001	….
	*P. rubi* DNA	0.10	0.22	0.67	1.0	….	0.10	0.22	0.63	….	0.26	0.21	0.22	….	0.14	0.22	0.49	….	0.15	0.22	0.49	….	0.39	0.21	0.06	….
	*P*	….	….	….	….	0.08	….	….	….	0.61	….	….	….	0.09	….	….	….	0.36	….	….	….	0.35	….	….	….	<0.001
	*R* ^2^	….	….	….	….	0.08	….	….	….	0.09	….	….	….	0.17	….	….	….	0.09	….	….	….	0.09	….	….	….	0.33
	ρ	….	….	….	….	….	….	….	….	−0.21	….	….	….	−0.85	….	….	….	….	….	….	….	….	….	….	….	….
	λ	….	….	….	….	….	….	….	….	….	….	….	….	….	….	….	….	−0.44	….	….	….	−1.02	….	….	….	−0.99
	AIC	….	….	….	….	206.40	….	….	….	208.14	….	….	….	206.19	….	….	….	207.56	….	….	….	207.53	….	….	….	189.57

**WA-1**	Intercept	0.48	0.14	0.001	….	….	0.51	0.25	0.04	….	−0.03	0.43	0.94	….	0.46	0.13	<0.001	….	0.48	0.13	<0.001	….	0.45	0.12	<0.001	….
	*P. penetrans* (soil)	0.09	0.12	0.42	1.0	….	0.10	0.11	0.38	….	−0.04	0.15	0.81	….	0.11	0.11	0.30	….	0.09	0.11	0.42	….	0.12	0.10	0.22	….
	*P. rubi* DNA	−0.01	0.07	0.85	1.0	….	−0.01	0.07	0.85	….	−0.006	0.07	0.93	….	−0.01	0.07	0.87	….	−0.01	0.07	0.88	….	−0.01	0.06	0.87	….
	*P*	….	….	….	….	0.71	….	….	….	0.88	….	….	….	0.69	….	….	….	0.72	….	….	….	0.78	….	….	….	0.65
	*R* ^2^	….	….	….	….	0.01	….	….	….	0.01	….	….	….	0.06	….	….	….	0.01	….	….	….	0.01	….	….	….	0.01
	ρ	….	….	….	….	….	….	….	….	−0.06	….	….	….	−0.17	….	….	….	….	….	….	….	….	….	….	….	….
	λ	….	….	….	….	….	….	….	….	….	….	….	….	….	….	….	….	−0.17	….	….	….	−0.21	….	….	….	−0.27
	AIC	….	….	….	….	134.05	….	….	….	136.03	….	….	….	137.17	….	….	….	135.92	….	….	….	135.97	….	….	….	135.84

**WA-2**	Intercept	0.80	0.49	0.11	….	….	0.61	0.73	0.40	….	1.06	1.35	0.43	….	0.59	0.43	0.18	….	0.69	0.47	0.14	….	0.70	0.46	0.13	….
	*P. penetrans* (soil)	0.47	0.22	0.04	1.0	….	0.45	0.22	0.04	….	0.34	0.21	0.10	….	0.52	0.20	0.01	….	0.53	0.21	0.01	….	0.50	0.21	0.02	….
	*P. rubi* DNA	0.26	0.14	0.06	1.0	….	0.24	0.14	0.08	….	0.08	0.14	0.58	….	0.36	0.12	0.003	….	0.26	0.13	0.05	….	0.31	0.13	0.02	….
	*P*	….	….	….	….	0.03	….	….	….	0.66	….	….	….	0.06	….	….	….	0.51	….	….	….	0.71	….	….	….	0.66
	*R* ^2^	….	….	….	….	0.12	….	….	….	0.12	….	….	….	0.29	….	….	….	0.13	….	….	….	0.12	….	….	….	0.12
	ρ	….	….	….	….	….	….	….	….	0.13	….	….	….	−0.82	….	….	….	….	….	….	….	….	….	….	….	….
	λ	….	….	….	….	….	….	….	….	….	….	….	….	….	….	….	….	−0.40	….	….	….	−0.24	….	….	….	−0.16
	AIC	….	….	….	….	190.27	….	….	….	192.08	….	….	….	183.53	….	….	….	191.85	….	….	….	192.14	….	….	….	192.08

a Independent variables were *P. rubi* DNA concentrations in roots and *P. penetrans* in soil. The *P* value represents the significance of the model at *P* < 0.05. *R*^2^ is the Nagelkerke pseudo-R-squared; ρ is a fitting parameter for SLM and SDM; λ is a fitting parameter for SEM, CAR, and SARMA. AIC was used to compare OLS, SLM, SDM, SEM, CAR, and SARMA models and determine the best model that fits the data for each field. The lower the AIC, the better the model.

AIC, Akaike information criterion; CAR, conditional autoregressive; OLS, ordinary least squares; *P*, probability; SARMA, spatial autoregressive moving average; SDM, spatial Durbin model; SE, standard errors; SEM, spatial error model; SLM, spatial lag model; VIF, variance inflation factor.

## Discussion

This study illustrates the widespread prevalence of *P. rubi* and *P. penetrans* within commercial red raspberry fields across the PNW. Both organisms were detected at high levels in all four fields sampled, but severe disease was more often associated with *P. rubi* than with *P. penetrans*. Across the four sampled fields, over 85% of the samples were positive for *P. rubi* DNA, and 69% of the root samples were positive for *P. penetrans*. These results further expand on previous studies that detected *P. rubi* and *P. penetrans* in over 20 commercial red raspberry fields sampled across Washington (Gigot et al., 2013; Weiland et al., 2018).

Spatial autocorrelation reflects processes that generate similarities between the values in nearby locations. This process is important for describing spillover effects, where, for example, a soilborne pathogen infestation in one location could influence other sites via active or passive secondary dispersal, leading to a disease outbreak across the field. If disease genesis is influenced by random factors or events in the fields, the spatial autocorrelation of disease incidence will tend to be low. In this study, there was significant spatial aggregation of disease severity, *P. rubi*, and *P. penetrans* in most of the sampled fields, reflecting similarities between field sampling locations and the prospect for spillover effects. Furthermore, soil texture and field elevation also had spatial dependency in their distributions across most of the sampled fields.

Our results are in line with previous studies where disease severity, *Phytophthora* spp., and *Pratylenchus* spp. were spatially clumped in infested fields across different cropping systems (Alby et al., 1983; Morgan et al., 2002; Gorny et al., 2020). *Phytophthora* spp. disease outbreaks have been reported to often result in patches of local infestation in the fields, which can be dispersed either in soil via surface water movement, rain splash, or by agriculture equipment and human activities (Larkin et al., 1995; Miller et al., 1997; Ristaino and Gumpertz, 2000). Larkin et al. (1995) showed that *Phytophthora capsici* was distinctly aggregated and not random in 4 of 7 pepper fields. Similarly, Widmark et al. (2007) found that *Phytophthora infestans*-infected plants were localized in six distinct foci within a potato field. *P. capsici* and *Phytophthora sojae* also had clumped distributions within intensively sampled pepper and soybean fields (Miller et al., 1997); however, the presence of the pathogens was also detected outside of disease centers at lower concentrations. Morgan et al. (2002) reported that *P. penetrans* were clumped in infested commercial potato fields, and different disease management strategies were found to influence the stability of the infestation foci.

This study enhanced our understanding of the connections between disease severity, soil texture, elevation, and *P. rubi* and *P. penetrans* distributions in infested fields using spatial autocorrelation models. In previous research, *P. rubi* frequency in raspberry fields was positively related to silt content (Gigot et al., 2013), and *Phytophthora* root rot has been reported to be more severe in low-lying areas where water collects (Duncan and Cowan, 1980; Erwin and Ribeiro, 1996; Weiland et al., 2018). However, in this study, greater *P. rubi* DNA in root and disease severity were not consistently influenced by soil texture or field elevation. This may be due to differences in several key factors among the fields. For example, soil texture varied greatly with the OR fields tending to have higher silt and clay contents than the WA fields. OR fields also had plants that were older than those in WA. Plants in OR-1 and OR-2 were 23 years old and 12 years old, respectively, whereas WA-1 and WA-2 had plants no older than 7 years. Finally, three of the fields were relatively flat, with little change in elevation: only OR-1 varied >10 m from one end of the field to the other. Still, higher levels of *P. rubi* and disease severity were not consistently found in the lowest part of the field. This is likely because most growers are irrigating their fields at levels that are more than enough to support infection by *P. rubi* (Weiland et al., 2018). The lack of a consistent response of *P. rubi* to elevation and soil texture makes it difficult to predict where disease foci will develop. Current soil fumigation and fungicide practices used by the industry do not adequately control *P. rubi* and need optimization (DeVetter et al., 2018; Weiland et al., 2018, 2024). Until effective treatments are identified and integrated with *P. rubi*-resistant cultivars, *Phytophthora* root rot will continue to be a yield-limiting disease.

*Pratylenchus* spp. have been reported to reach higher population densities and move more easily in coarse-texture soils than in finer-texture soils (Townsend and Webber, 1971; Blair et al., 1999). However, in this study, greater *P. penetrans* densities in soil were not consistently influenced by soil texture or elevation, although some significant influences were observed for WA-1. Our mixed results may be due to differences in key field factors such as planting ages, heterogeneity in field landscape features, and agronomic practices. To further understanding of the factors that determine the distribution of *P. rubi* and *P. penetrans*, it may be useful to conduct a similar study on a larger number of fields with more similarities in age, elevation, and soil type.

In PNW red raspberry fields, *P. penetrans* and *P. rubi* share an environment in which they may interact. In this study, disease severity was often greater in areas where there were higher concentrations of *P. rubi* DNA in the roots, but lower densities of *P. penetrans* in the soil for OR-1 and WA-1. However, we observed that high *P. rubi* DNA concentrations in roots and high *P. penetrans* densities in soil significantly increased disease severity in WA-2, and the disease severity map overlapped with the distribution maps of these two soilborne pathogens. However, there were complexities and inconsistencies in determining whether only *P. rubi* caused the disease symptoms or whether both *P. rubi* and *P. penetrans* contributed to high disease severity in raspberry plants. Our mixed results may be partly due to the distribution of soil texture in the field. For example, disease severity, *P. rubi* DNA concentration in root, *P. penetrans* densities in soil, sand, and silt maps relatively overlapped, where high disease severity, high *P. rubi* DNA concentrations in root, high *P. penetrans* densities in soil, high sand, and low clay contents relatively coincided. Nematodes are strictly obligate parasites requiring living tissues to reproduce and proliferate while moving easily in coarse-texture soils. It is possible if *P. rubi* rotted all the roots, *P. penetrans* densities might be depressed in the soil as they move to other parts of the field where *P. rubi* has not destroyed all the roots yet. Weiland et al. (2024) suggested that *P. rubi*, *P. penetrans*, and *Verticillium dahliae* potentially cause a disease complex in red raspberry. However, there is no definitive evidence to suggest that *P. rubi* and *P. penetrans* interact to cause worse disease. Additionally, we found that *P. rubi* DNA concentration had no significant influence on *P. penetrans* densities in the roots and that only *P. penetrans* densities in soil had a significant positive effect on nematode densities in raspberry roots. In most cases where the two organisms co-occur, it appears that *P. rubi* is the main agent causing the most root disease (Vrain and Pepin, 1989; Gigot et al., 2013; Weiland et al., 2018).

Based on this study, it would be difficult to move away from full field soil fumigation to control *P. rubi* and *P. penetrans* when establishing a new planting due to the widespread distribution of these pathogens within most fields. Though no universal factors can be concluded to indicate the risk of *P. rubi* and *P. penetrans* to raspberry production, knowing where the pathogen and disease are located within a field is still helpful information to a grower to indicate where management practices can be targeted.

## Supplementary Materials

**Table S1:** Global Moran’s I test for spatial autocorrelation in the residuals for *Phytophthora rubi* DNA concentrations in roots, *Pratylenchus penetrans* densites in root and soil regressed against soil texture (sand, silt, and clay), and field elevation in four commercial red raspberry production fields in Oregon (OR-1 and OR-2) and Washington (WA-1 and WA-2).

**Field**	**Predicted**	**Moran’s I coefficient^a^**	***P*-value^b^**
**OR-1**	*P. rubi* DNA in root	−0.018	0.29
	*P. penetrans* in root	−0.026	0.39
	*P. penetrans in soil*	−0.030	0.45
**OR-2**	*P. rubi* DNA in root	−0.011	0.39
	*P. penetrans* in root	−0.016	0.44
	*P. penetrans in soil*	0.046	0.03
**WA-1**	*P. rubi* DNA in root	−0.017	0.36
	*P. penetrans* in root	−0.001	0.19
	*P. penetrans in soil*	0.100	<0.001
**WA-2**	*P. rubi* DNA in root	0.064	<0.001
	*P. penetrans* in root	−0.060	0.78
	*P. penetrans in soil*	0.030	0.01

a Global Moran’s I coefficient values range from −1 to 1, where a value <0 indicates negative spatial autocorrelation and a value >0 indicates positive spatial autocorrelation.

b The *P*-values indicate the level of significance at *P* < 0.05 for each coefficient.

**Table S2:** Global Moran’s I test for spatial autocorrelation in the residuals for visual disease rating regressed against *Phytophthora rubi* DNA concentrations in roots and *Pratylenchus penetrans* densities in roots and soil in four commercial red raspberry production fields in Oregon (OR-1 and OR-2) and Washington (WA-1 and WA-2).

**Field**	**Moran’s I coefficient^a^**	***P*-value^b^**
**OR-1**	0.13	<0.001
**OR-2**	−0.01	0.41
**WA-1**	0.03	0.05
**WA-2**	0.19	<0.001

a Global Moran’s I coefficient values range from −1 to 1, where a value <0 indicates negative spatial autocorrelation and a value >0 indicates positive spatial autocorrelation.

b The *P*-values indicate the level of significance *P* < 0.05 for each coefficient.

**Table S3:** Global Moran’s I test for spatial autocorrelation in the residuals for *Pratylenchus penetrans* densities in root regressed against *P. penetrans* densities in soil and *Phytophthora rubi* DNA concentrations in roots in four commercial red raspberry production fields in Oregon (OR-1 and OR-2) and Washington (WA-1 and WA-2).

**Field**	**Moran’s I coefficient^a^**	***P*-value^b^**
**OR-1**	−0.01	0.39
**OR-2**	−0.03	0.66
**WA-1**	−0.01	0.36
**WA-2**	−0.02	0.45

a Global Moran’s I coefficient values range from −1 to 1, where a value <0 indicates negative spatial autocorrelation and a value >0 indicates positive spatial autocorrelation.

b The *P*-values indicate the level of significance *P* < 0.05 for each coefficient.
